# SARS-CoV-2 N protein enhances the anti-apoptotic activity of MCL-1 to promote viral replication

**DOI:** 10.1038/s41392-023-01459-8

**Published:** 2023-05-09

**Authors:** Pan Pan, Weiwei Ge, Zhiwei Lei, Wei luo, Yuqing Liu, Zhanwen Guan, Lumiao Chen, Zhenyang Yu, Miaomiao Shen, Dingwen Hu, Qi Xiang, Wenbiao Wang, Pin Wan, Mingfu Tian, Yang Yu, Zhen Luo, Xulin Chen, Heng Xiao, Qiwei Zhang, Xujing Liang, Xin Chen, Yongkui Li, Jianguo Wu

**Affiliations:** 1 The First Affiliated Hospital of Jinan University, 510632 Guangzhou, China; 2 Foshan Institute of Medical Microbiology, 528315 Foshan, China; 3 Guangdong Provincial Key Laboratory of Virology, Institute of Medical Microbiology, Jinan University, 510632 Guangzhou, China; 4 State Key Laboratory of Virology, College of Life Sciences, Wuhan University, 430072 Wuhan, China; 5 The First People’s Hospital of Foshan, 528315 Foshan, China

**Keywords:** Infectious diseases, Microbiology, Infection

## Abstract

Viral infection in respiratory tract usually leads to cell death, impairing respiratory function to cause severe disease. However, the diversity of clinical manifestations of SARS-CoV-2 infection increases the complexity and difficulty of viral infection prevention, and especially the high-frequency asymptomatic infection increases the risk of virus transmission. Studying how SARS-CoV-2 affects apoptotic pathway may help to understand the pathological process of its infection. Here, we uncovered SARS-CoV-2 imployed a distinct anti-apoptotic mechanism via its N protein. We found SARS-CoV-2 virus-like particles (trVLP) suppressed cell apoptosis, but the trVLP lacking N protein didn’t. Further study verified that N protein repressed cell apoptosis in cultured cells, human lung organoids and mice. Mechanistically, N protein specifically interacted with anti-apoptotic protein MCL-1, and recruited a deubiquitinating enzyme USP15 to remove the K63-linked ubiquitination of MCL-1, which stabilized this protein and promoted it to hijack Bak in mitochondria. Importantly, N protein promoted the replications of IAV, DENV and ZIKV, and exacerbated death of IAV-infected mice, all of which could be blocked by a MCL-1 specific inhibitor, S63845. Altogether, we identifed a distinct anti-apoptotic function of the N protein, through which it promoted viral replication. These may explain how SARS-CoV-2 effectively replicates in asymptomatic individuals without cuasing respiratory dysfunction, and indicate a risk of enhanced coinfection with other viruses. We anticipate that abrogating the N/MCL-1-dominated apoptosis repression is conducive to the treatments of SARS-CoV-2 infection as well as coinfections with other viruses.

## Introduction

Coronavirus (CoV) can infect the respiratory, alimentary and central nervous systems of humans and other mammal animals.^1^ The outbreaks of the severe acute respiratory syndrome (SARS) in 2002 and the Middle East respiratory syndrome (MERS) in 2012 were caused by infections of SARS-CoV and MERS-CoV respectively, and were associated with animal-to-human transmission.^2^ The Coronavirus Disease 2019 (COVID-19) resulting from SARS-CoV-2 infection is extremely pathogenic and contagious.^3,4^ Beside the asymptomatic infections, the COVID-19 patients suffer from fever, dry cough, body aches and fatigue, and the manifestations may gradually develop into dyspnea, viral pneumonia, multiple organ damage and failure in critically ill patients, and even death.^5^ The SARS-CoV-2 infection has caused significant harm to human health and global economy, and it is urgent to pay attention to reducing its transmission. The pathogenic mechanism of SARS-CoV-2 infection needs to be urgently clarified,which is crucial to the development of effective therapeutic strategies.

The asymptomatic fraction of infection, which is the proportion of infected individuals who never develop, perceive, and report symptoms, usally exists and varies widely in the infections of different pathogens.^6^ The asymptomatic fraction of SARS-CoV-2 infection seems to be sizable, which includes presymptomatic (virus is detectable ahead of symptom onset) and asymptomatic (virus is detectable but symptoms never emerge).^7^ Epidemiological research reveal that asymptomatic individuals also can produce infectious viruses and transmit them to others, supporting that the presymptomatic and asymptomatic infectious statuses are important. SARS-CoV-2 infection is primarily diagnosed through detecting viral RNA by real-time PCR. The cycle threshold (Ct) values by real-time PCR can reflect infectiousness, as lower Ct values mean higher viral load.^8^ Clinical studies have found that presymptomatic (Ct value rang 15-38)^9^ and asymptomatic (Ct value rang 14-40)^9–13^ infected individuals also have high viral loads, and infectious viruses can be isolated even under the condition of high CT value at 34.^9^ It is an important question how an asymptomatic infected person can have such a high viral load without any clinical symptom. Previous studies had found that the asymptomatic SARS-CoV-2 individuals develop a protective mechanism by a balanced antiviral cellular immunity, which not only allows viral replication but also avoids pathological damage.^14^ Besides immune function, host utilizes cell apoptosis for antiviral defense, which can reduce viral replication by removing infected cells. Moreover, the apoptosis of infected cells aviods overactivating immune and inflammatory responses to virus.^15^ Therefore, studying cell apoptosis during the infection of SARS-CoV-2 is of great significance.

Apoptosis, an important physiological process of the host, is tightly regulated and represents an active and orderly way of cell death. Apoptosis is involved in various physiological processes and is of great significance for maintaining the ontogeny and homeostasis of organisms. The apoptosis process is divided into endogenous and exogenous apoptosis.^16^ The proteins of B-cell lymphoma 2 (BCL-2) family control the initiation of the apoptotic program by regulating the mitochondrial pathway in endogenous apoptosis.^17^ Myeloid cell leukemia-1 (MCL-1) belongs to the anti-apoptotic BCL-2 family.^18^ MCL-1 is different from other family members because its half-life is very short. MCL-1 acts as anti-apoptotic protein by binding and inhibiting BCL2 Antagonist/Killer (Bak) and BCL2-Associated X (Bax), both of which are pro-apoptotic proteins.^19^ The stimulation of death receptors by their ligands also activates extrinsic apoptotic pathways.^20^ The cleavage and activation of the key protein Caspase-3 (Casp-3) play a critical role in the two apoptotic pathways.^17,20^ Upon virus infection, the host cells activate the apoptotic pathway to eliminate immature virus particles, but the virus is able to evolve and develop a mechanism to inhibit apoptosis to ensure its reproduction in the early stage of infection. Previous studies reported that SARS-CoV initiates an extrinsic apoptosis pathway through the interaction of its S protein with cell membrane death receptor^21^ and M protein to promote apoptosis through the Pyruvate Dehydrogenase Kinase 1(PDK1)- PDZ Binding Kinase (PBK)/ Protein Kinase B (AKT) pathway,^22^ while its E protein inhibits apoptosis by regulating stress response.^22,23^ Current studies have revealed that SARS-CoV-2 infection can activate apoptosis by regulating the Caspase-8/3 pathway^24^ and that the ORF3a of SARS-CoV-2 may promote apoptosis.^25^ However, the function of other proteins encoded by this virus in regulating cell apoptosis and viral replication remain unknown.

The N protein of SARS-CoV-2 contains functional domains with RNA-binding activity at its N- and C-terminal, which can encapsulate the viral genomic RNA and is involved in virus replication.^26^ Previous studies revealed that the N protein could activate NLRP3 (NOD-like receptor thermal protein domain associated protein 3) inflammasome, induce cytokine storm, and cause lung injury.^27^ The N protein also causes acute kidney injury *via* interfering with the cell cycle^28^ and inhibits interferon-β (IFN-β) production by suppressing RIG-I (retinoic acid-inducible gene I) signaling.^29^ In addition, N protein promotes viral replication by impairing stress granule formation^30^ and suppresses antiviral immune response by regulating Mitochondrial antiviral-signaling protein (MAVS) activity.^31^ However, the molecular mechanisms by which N regulates cell apoptosis and viral replication remain undetermined.

The public health restrictions to control the SARS-CoV-2 spread have effectively reduced the transmissions of other kinds of endemic respiratory viruses. However, as many countries don’t use such measures, the world will face a higher risk of co-infections of SARS-CoV-2 and other respiratory viruses. In fact, a clinical analysis revealed that co-infection of SARS-CoV-2 and influenza virus resulted in more severe clinical symptoms and higher mortality rate than separate infections.^32^ Other results showed that influenza virus enhanced SARS-CoV-2 infection through promoting Angiotensin-converting enzyme 2 (ACE2) expression.^33^ However, the influence of SARS-CoV-2 on the infection of influenza virus remain unknown. In consideration of the wide SARS-CoV-2 transmission, it is of critical importance to explore the involved mechanism during co-infection with other viruses.

Here, we report that the N protein suppresses cell apoptosis by regulating the anti-apoptotic protein MCL-1 function. We found N protein specifically interacted with MCL-1 and promoted MCL-1 deubiquitination to extend its half-life. Notably, the deubiquitinating enzyme USP15 was required for N-mediated MCL-1 deubiquitination. Interestingly, N protein promoted the replication of influenza A virus (IAV), Dengue virus (DENV-2) and Zika virus (ZIKV), by enhancing MCL-1 protein level and exacerbated the death of IAV-infected mice, while such regulations were blocked by S63845, a specific inhibitor of MCL-1.^34^ Altogether, this work revealed a distinct molecular mechanism via which N protein promoted virus replication by regulating the apoptosis pathway and provided new insights into the viral replication regulation, co-infection and pathogenesis.

## Results

### SARS-CoV-2 N protein represses cell apoptosis

Although cell death pathways in SARS-CoV-2 infection have been characterized,^35–38^ the mechanism via which this virus regulates cell apoptosis remains mainly unclear. Here, we initially studied how SARS-CoV-2 affected cell apoptosis by analyzing a previously reported cell culture system for replicating competent SARS-CoV-2 virus-like-particles (SARS-CoV-2-trVLP)^39^ in which SARS-CoV-2 N gene was replaced by the GFP gene (GFP/ΔN). The complete life cycle of SARS-CoV-2 was achieved and exclusively confined in the cells stably expressing the N protein.^39^ Caco-2 cells were transfected with different doses of N-expressing plasmid, then infected with SARS-CoV-2-GFP/ΔN trVLP. Notably, the basal levels of cell apoptosis were attenuated by N protein in a dose-dependent manner in condition of SARS-CoV-2-trVLP infection (Supplementary Fig. 1a and 2a) based on flow cytometry analyses of apoptosis.

**Fig. 1:**
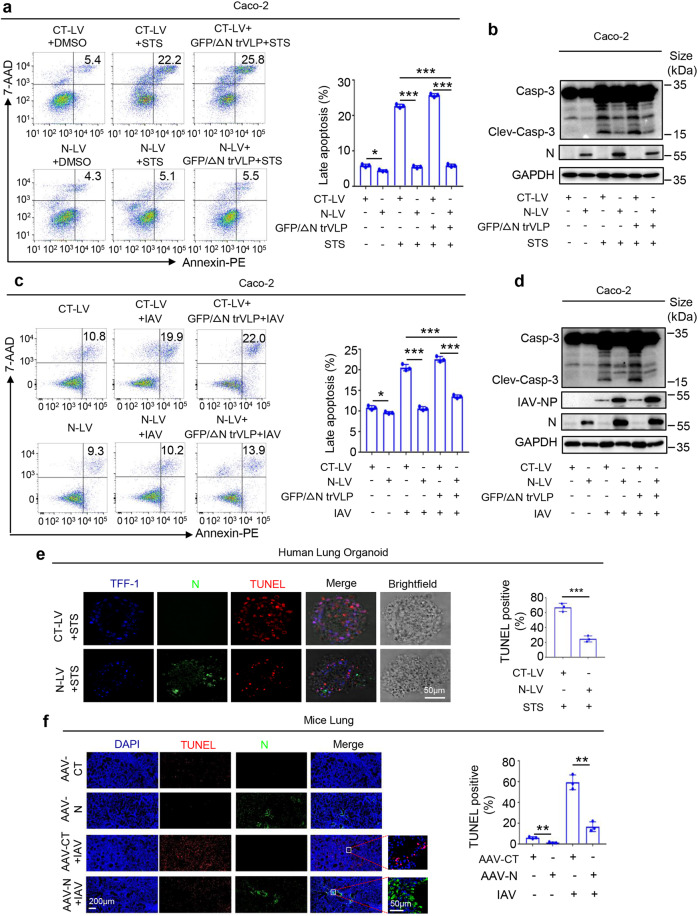
SARS-CoV-2 N protein represses cell apoptosis. **a**, **b** Caco-2 cells were stably infected with Lentivirus-CT (CT-LV) or Lentivirus-N (N-LV), and CT-LV and N-LV cells pre-infected with SARS-CoV-2-trVLP (MOI = 0.5) for 24 h, then CT-LV and N-LV cells stimulated with 5 μM Staurosporine for another 4 h. Cell apoptosis level was detected by Flow cytometry (**a**). Cell lysates were analyzed by immunoblotting (**b**). **c**, **d** Caco-2 cells were stably infected with Lentivirus-CT (CT-LV) or Lentivirus-N (N-LV), and CT-LV and N-LV cells pre-infected with SARS-CoV-2-trVLP (MOI = 0.5) for 12 h, then CT-LV and N-LV cells infected with influenza virus (PR8 strain, MOI = 0.1) for another 24 h. The cell apoptosis levels were detected by Flow cytometry (**c**). Cell lysates were analyzed by immunoblotting (**d**). **e** Human Lung organoids were infected with Lentivirus-CT or Lentivirus-N and then stimulated with 5 μM Staurosporine for 4 h. TFF-1 (blue), Flag-SARS-CoV-2-N (green), and TUNEL (red) were then visualized with confocal microscopy (left). Scale bar, 50 μm (**e**). The TUNEL positive percent were statistics (right). **f** C57BL/6 genetic mice were given tail vein injection with 300 μl containing 3 × 10^11^ vg of AAV-Lung-EGFP (*n* = 8) or AAV-Lung-N (*n* = 8). After three weeks since the intranasal injection with the influenza virus (PR8 strain, 1LD50), another untreated AAV-Lung-EGFP or AAV-Lung-N infected mice were prepared as the blank group (*n* = 4). Six days after infection, two mice were euthanized, and the lung tissues were collected. Histoimmunofluorescence analysis of TUNEL (red) and SARS-CoV-2-N (green) in the lungs (**f**, left). Scale bar, 200 μm. The TUNEL positive percent were statistics (**f**, right). CT-LV means CT-lentivirus and N-LV means N-lentivirus (**a**–**e**). AAV-CT means AAV-Lung-EGFP (**f**). GFP/ΔN trVLP means SARS-CoV-2-GFP/ΔN trVLP (**a**–**d**). STS means Staurosporine (**a**, **b**, **e**). Data are representative of three independent experiments, with one representative shown. Error bars indicated the SD of technical triplicates. The values represent mean ± SEM. *P ≤ 0.05, **P ≤ 0.01, ***P ≤ 0.001, two-tailed Student’s *t* test

The apoptosis inhibitory effects of N protein were further determined by two approaches. Caco-2 cells were infected with the Lentivirus carrying N gene (N-Lentivirus) to generate cells stably expressing N protein (Caco-2-N cells) or infected with negative control Lentivirus (CT-Lentivirus) to generate control cells (Caco-2-CT cells). First, Caco-2-CT or Caco-2-N cells were treated with Staurosporine (STS), a specific activator of the apoptosis pathway^40^ or infected with SARS-CoV-2-GFP/ΔN trVLP, then treated with STS. Compared with CT-LV + DMSO group, N-LV + DMSO group slightly inhibited apoptosis; markedly, cell apoptosis was stimulated by STS in Caco-2-CT cells, but significantly reduced in Caco-2-N cells; meanwhile, cell apoptosis was significantly induced by STS in Caco-2-CT cells infected with SARS-CoV-2-GFP/ΔN trVLP, while STS-activated cell apoptosis was significantly repressed in Caco-2-N cells infected with intact SARS-CoV-2-trVLP (Fig. 1a and Supplementary Fig. 2b). Cleaved Casp-3 was significantly activated by STS in Caco-2-CT cells or Caco-2-CT cells infected with SARS-CoV-2-GFP/ΔN trVLP, but STS-induced cleaved Casp-3 was both inhibited in Caco-2-N cells or Caco-2-N cells infected with intact SARS-CoV-2-trVLP (Fig. 1b). Second, Caco-2-CT or Caco-2-N was infected with influenza A virus (IAV)^41^ or infected with SARS-CoV-2-GFP/ΔN trVLP, then infected with IAV. Notably, compared with CT-LV group, N-LV group slightly inhibited apoptosis; markedly, cell apoptosis was significantly induced upon IAV infection in Caco-2-CT cells, but remarkly suppressed upon IAV infection in Caco-2-N cells. Similarly, We also observed that cell apoptosis was significantly induced upon IAV infection in Caco-2-CT cells infected with SARS-CoV-2-GFP/ΔN trVLP, but remarkly suppressed upon IAV infection in Caco-2-N cells infected with intact SARS-CoV-2-trVLP (Fig. 1c and Supplementary Fig. 2c). Cleaved Casp-3 was significantly activated upon IAV infection in Caco-2-CT cells or Caco-2-CT cells infected with SARS-CoV-2-GFP/ΔN trVLP, but IAV-induced cleaved Casp-3 was both inhibited in Caco-2-N cells or Caco-2-N cells infected with intact SARS-CoV-2-trVLP (Fig. 1d).

Next, we investigated which protein of SARS-CoV-2 was involved in the repression of cell apoptosis. Plasmids carrying SARS-CoV-2 genes encoding three structural proteins (N, M, and E) and one accessory protein (ORF3a) were transfected into A549, HULEC and THP-1 cells. The results showed that cleaved Casp-3 was repressed by N and E protein in A549 cells, by N protein in HULEC cells or by N and 3a protein in THP-1 cells. Only N protein could inhibite cells apoptosis in all the above cells (Supplementary Fig. 1b). Thus, the effect of N protein on cell apoptosis was further determined. THP-1 and A549 cells were infected with N-Lentivirus and CT-Lentivirus to generate two cell lines that stably expressing N-Lentivirus (THP-1-N cells and A549-N cells) and two cell lines stably expressing CT-Lentivirus (THP-1-CT cells and A549-CT cells). THP-1-N and THP-1-CT cells were subsequently differentiated into macrophages. All cells were then treated with STS. The results showed that cleaved Casp-3 was significantly induced by STS, but such induction was remarkably repressed by N protein in the cells (Supplementary Fig. 1c). Notably, CCK8 analysis results indicated that cell viabilities were facilitated by N protein in the treated cells (Supplementary Fig. 1d). We also noticed that STS significantly repressed cell viabilities, but STS-mediated inhibitions were suppressed by the N protein (Supplementary Fig. 1d). Further, cell apoptosis was induced by STS, but STS-mediated induction was repressed in A549-N cells (Supplementary Fig. 1e and 2d), suppressed in THP-1-N differentiated macrophages (Supplementary Fig. 1f and 2e), and attenuated by N protein in a dose-dependent manner (Supplementary Fig. 1g and 2f). Moreover, the effects of N protein on cell apoptosis were determined in human lung organoids, which were examined and photographed under a microscope (Supplementary Fig. 3a). The levels of flat type I alveolar epithelial cell (AT1) related markers (AQP5, HOPX, and AGER) and cuboidal type II alveolar epithelial cell (AT2) related markers (SFTPA, SFTPB, SFTPC, SFTPD, ABAC3 and LAMP3)^42^ in the lung organoids were detected (Supplementary Fig. 3b). Immunofluorescence experiments demonstrated that N protein (in green) was expressed and co-localized with the lung epithelial cell marker TFF-1^43^ (in blue) in the lung organoids infected with Lentivirus-N (Supplementary Fig. 3c). We also noticed that STS induced cell apoptosis (TUNEL positive), but STS-mediated induction was repressed by N protein in the lung organoids (Fig. 1e). Lastly, the effects of N protein on cell apoptosis was determined in an AAV-Lung-N C57BL/6 mice.^27^ C57BL/6 mice were injected via tail vein with AAV-Lung-N or AAV-Lung-EGFP to generate AAV-Lung-N C57BL/6 mice and AAV-Lung-EGFP C57BL/6 mice, which were then infected with IAV through nasal drops. TUNEL positive cells were significantly induced upon IAV infection, while IAV-induced positive cells were significantly reduced by N protein (Fig. 1f). Together, these results demonstrate that SARS-CoV-2 N protein represses cell apoptosis in cultured cells, lung organoids and C57BL/6 mice.

In addition to cell apoptosis, the cell death pathways also include cell pyroptosis, cell autophagy, and cell iron death.^44^ Next, we assessed the impact of N protein on other cell death pathways. THP-1-CT or THP-1-N cells were subsequently differentiated into macrophages and incubated with Nigericin (NG), a specific NLRP3 inflammasome activator.^45^ We noted that the cleaved Gasdermin D (GSDMD), a marker molecule of the pyroptosis pathway,^46^ was induced by NG, and the N protein could further evelate the Nigericin-stimulated cleaved GSDMD levels (Supplementary Fig. 3d). This result was consistant with our previous report,^27^ which indicated that N protein promotes the level of cleaved Caspase-1 through activating the NLRP3 inflammasome. In addition, stable A549 cells and stable THP-1 differentiated macrophages were stimulated with Erastin (ERa), an activator for the iron death pathway.^47^ The levels of NADPH oxidase 1 (NOX1) and glutathione peroxidase 4 (GPX4), two marker molecules of iron death pathway,^47^ were not influenced by N protein in A549 cells and THP-1 macrophages (Supplementary Fig. 3e) treated with or without Erastin. Moreover, stable THP-1 differentiated macrophages and stable A549 cells were treated with Rapamycin (Rap), an activator for the autophagy pathway.^48^ The levels of microtublule associated protein 1 light chain 3 (LC3) and autophagy protein p62 (P62), two marker molecules of autophagy pathway,^48^ were not affected by N protein in in stable A549 cells or in stable THP-1 macrophages (Supplementary Fig. 3f) stimulated with or without Rapamycin.

Compared with SARS-CoV-2 original strain (Genebank NO. MN908947.3) N protein, Delta strain (Genebank NO. OP801647.1) has amino acid mutations at three sites (D63G、R203M and D377Y), Omicron strain(Genebank NO. OQ244249.1) has amino acid mutations at three sites (P13L、RG203/204KR) (Supplementary Fig. 4a, b). The A549 cells or THP-1 cells were respectively transfected with WT-N, Dlta-N and Omic-2 N protein, then treated with STS, the result showed that like WT-N protein, Cleaved Casp-3 was also inhibited by Dlta-N and Omic-N protein (Supplementary Fig. 4c, d).

Taken together, SARS-CoV-2 N protein activates cell pyroptosis, represses cell apoptosis, but has no effect on cell autophagy or iron death pathways.

### N represses apoptosis by regulating MCL-1

Here, we investigated the mechanism via which N protein repressed cell apoptosis. The results showed that the mRNA levels of pro-apoptosis genes (Bid, Bam, Bak and Bax), Casp-8, Casp-3 and anti-apoptosis genes (BCL-XL, BCL-W, BCL-2 and MCL-1) were relatively unaffected by N protein in treated cells (Supplementary Fig. 5a, b). Notably, N protein had no significant effect on Bim, Bak, Casp-8, Casp-3, BCL-XL and BCL-W proteins but could promote the anti-apoptosis protein MCL-1 production (Supplementary Fig. 5c, d). These results proved that N protein played a positive role in regulating MCL-1 protein production.

**Fig. 2:**
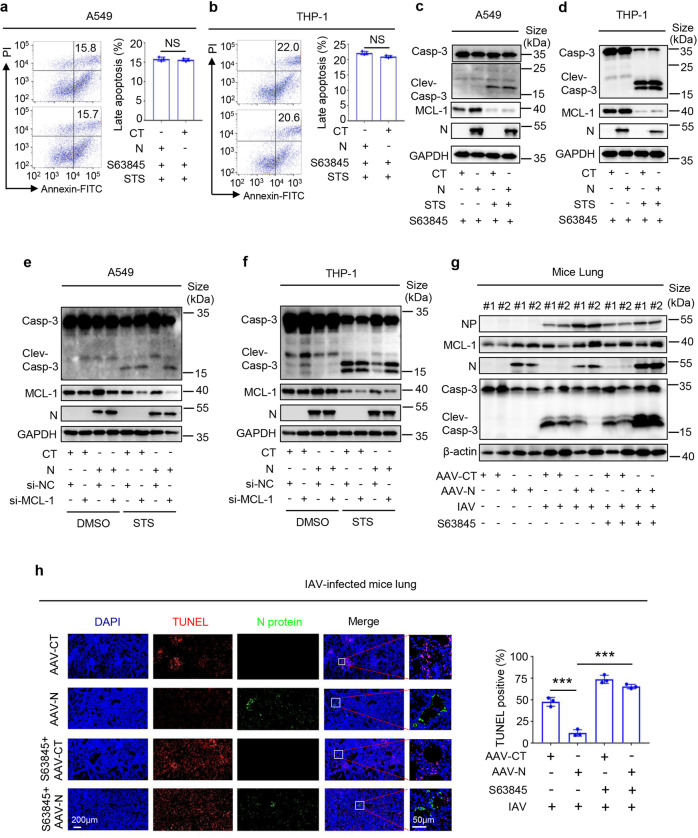
N represses apoptosis by regulating MCL-1. **a**–**d** A549 cells (**a**, **c**) and THP-1 cells (**b**, **d**) were stably infected with Lentivirus-CT or Lentivirus-N. THP-1 cells were differentiated into macrophages. They were pre-treated with 3 μM MCL-1 inhibitor S63845 for 4 h, then stimulated with 5 μM Staurosporine or DMSO for 4 h. The cell apoptosis level was detected by Flow cytometry. **a**, **b** Cell lysates were analyzed by immunoblotting (**c**, **d**). **e**, **f** A549 cells (**e**) and THP-1 cells (**f**) were stably infected with Lentivirus-CT or Lentivirus-N, THP-1 cells were differentiated into macrophages, and the cells were separately transfected with si-NC or si-MCL-1 (50 nM) for 48 h, then stimulated with 5 μM Staurosporine or DMSO for 4 h. Cell lysates were analyzed by immunoblotting. **g**, **h** C57BL/6 genetic background mice were given tail vein injection with 300 μl containing 3 × 10^11^ vg of AAV-Lung-EGFP (*n* = 20) or AAV-Lung-N (*n* = 20). After three weeks, they were pre-treated with MCL-1 specific inhibitor S64845 (12.5 mg/kg) by intraperitoneal injection for 90 min and infected with influenza virus (PR8 strain, 1LD50) by intranasal injection, followed by treatment with S64845 (12.5 mg/kg) on the fourth day after influenza virus infection (*n* = 8), or the same volume solvent (50% PEG300 plus 50% PBS) as a control group (*n* = 8). Another untreated AAV-Lung-EGFP or AAV-Lung-N infected mice were used as the blank group (*n* = 4). Six days after infection, two mice were euthanasia, and the lung tissues were collected. The indicated proteins were measured by western blot (**g**). Histoimmunofluorescence analysis of TUNEL (red) and SARS-CoV-2-N (green) in the lung (**h**, left). Scale bar, 200 μm. The TUNEL positive percent were statistics (**h**, right). CT means CT-lentivirus (**a**–**f**). AAV-CT means AAV-Lung-EGFP (**g**, **h**). Data are representative of three independent experiments, and one representative is shown. Error bars indicate the SD of technical triplicates. The values represent mean ± SEM. *P ≤ 0.05, **P ≤ 0.01, ***P ≤ 0.001, two-tailed Student’s *t* test

Next, the cells were pre-incubated with S63845, a specific inhibitor for MCL-1 protein,^34^ then stimulated with STS. Flow cytometry results showed that STS-induced cell apoptosis was not influenced by N protein in cells pre-treated with S63845 (Fig. 2a, b and Supplementary Fig. 2g, h). Cleaved Casp-3 was induced by STS but not affected by N protein, while MCL-1 protein was inhibited by STS but not influenced by N protein in cells pre-treated with S63845 (Fig. 2c, d). In addition, the role of MCL-1 in N protein-mediated repression of cell apoptosis was determined using the small interfering RNA (siRNA) approach. The A549 or THP-1 cells were transfected with three siRNAs targeted to the MCL-1 gene (siMCL-1-1、siMCL-1-2 and siMCL-1-3) and the negative control siRNA (siNC), the results showed that MCL-1 protein was significantly inhibited by siMCL-1-1 both in A549 and THP-1 cells (Supplementary Fig. 6a, b).The A549-CT and A549-N cells or THP-1-CT or THP-1-N cells were respectively transfected with siMCL-1 and siNC, then treated with STS. We observed that MCL-1 protein was attenuated by siMCL-1-1 (Fig. 2e, f), while STS enhanced cleaved Casp-3 was suppressed by N protein, but further restored by siMCL-1-1 both in A549-N or THP-1-N cells (Fig. 2e, f).

The role of MCL-1 in cell apoptosis mediated by N was further determined in C57BL/6 mice. The mice were infected with AAV-Lung-EGFP and AAV-Lung-N to construct AAV-Lung-EGFP-C57BL/6 mice and C57BL/6 AAV-Lung-N-C57BL/6 mice. The mice were pre-treated with S64845, then infected with IAV. The results showed that cleaved Casp-3 was reduced by AAV-N in IAV-infected mice lungs but was promoted by AAV-N in IAV-infected mice lungs pre-treated with S63845 (Fig. 2g), indicating that S63845 could restore the N-mediated inhibition of cleaved Casp-3. Immunohistochemical fluorescence analyses showed that cell apoptosis (TUNEL positive cell) was repressed by AAV-N in IAV-infected mice lungs but promoted by AAV-N in IAV-infected mice lungs pre-treated with S63845 (Fig. 2h), suggesting that S63845 could restore the N-mediated repression of cell apoptosis.

SARS-CoV N protein has 89.7% homology with SARS-CoV-2 N protein (Supplementary Fig. 7a). The A549 cells were respectively transfected with CoV-N and CoV-2 N protein, then treated with STS, the result showed that like CoV-2 N protein, Cleaved Casp-3 was also inhibited by CoV-N protein (Supplementary Fig. 7b). Next, the A549 cells were transfected with CoV-N protein, pre-treated with S63845, then stimulated with STS. We observed that STS enhanced cleaved Casp-3 was inhibited by CoV-N protein; however, further promoted by S63845, indicating that S63845 could restore the CoV-N-mediated inhibition of apoptosis (Supplementary Fig. 7c).

Collectively, these results demonstrated that MCL-1 was required for the repression of cell apoptosis mediated by N and suggested that the SARS-CoV-2 N protein repressed cell apoptosis by regulating the MCL-1 protein.

### N protein interacts with MCL-1 protein

**Fig. 3:**
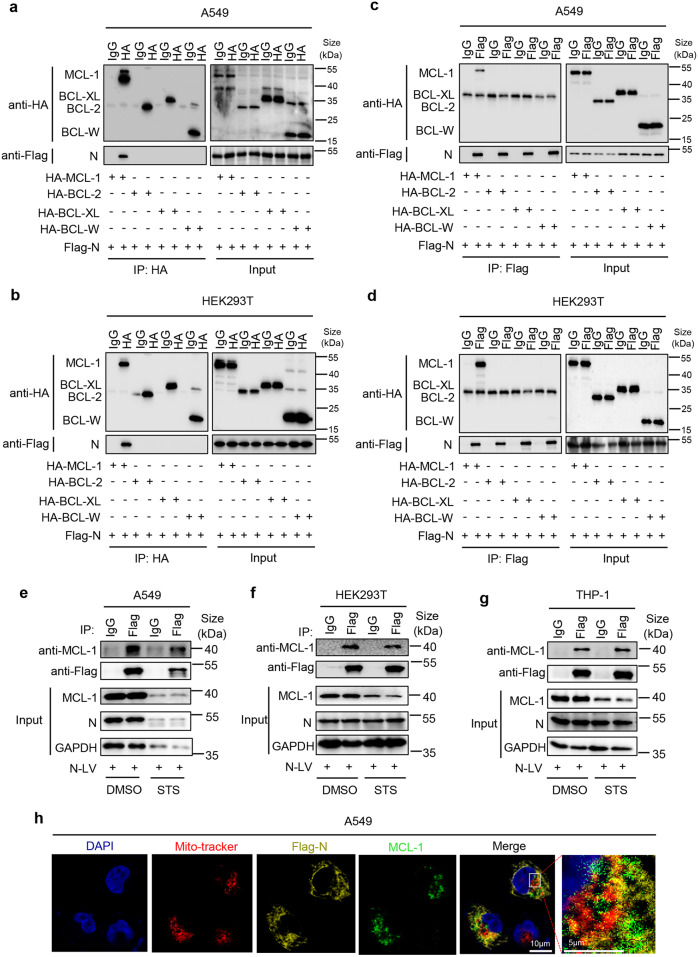
N protein interacts with MCL-1 protein. **a**–**d** A549 cells or HEK293T cells were co-transfected with Flag-SARS-CoV-2-N and HA-MCL-1, HA-BCL-2, HA-BCL-XL, or HA-BCL-W. Cell lysates were immunoprecipitated using an anti-HA antibody (**a**, **b**) or anti-Flag antibody (**c**, **d**) and analyzed using anti-Flag and anti-HA antibodies. Cell lysates (40 μg) were used as Input. **e**–**g** A549 cells (**e**), HEK293T cells (**f**) or THP-1 cells (**g**) were stably infected with Lentivirus-N, THP-1 cells were differentiated into macrophages, then stimulated with 5 μM Staurosporine or DMSO for 4 h. Cell lysates were immunoprecipitated using an anti-Flag antibody and analyzed using anti-Flag and anti-MCL-1 antibody. Cell lysates (40 μg) were used as Input. **h** A549 cells were stably infected with Lentivirus-N, Nucleus marker DAPI (blue), Flag-SARS-CoV-2-N (green), and MCL-1 (red) were then visualized with confocal microscopy. Scale bar, 10 μm. N-LV means N-lentivirus (**e**–**g**). Data are representative of three independent experiments, and one representative is shown

The mechanism by which MCL-1 regulates N protein-mediated cell apoptosis was explored. We initially determined whether N could interact with anti-apoptosis proteins. Co-immunoprecipitation (Co-IP) assays proved that only MCL-1 could interact with N, while BCL-XL, BCL-W, and BCL-2 failed to interact with N (Fig. 3a, b). Reciprocal Co-IP assays confirmed that N interacted with MCL-1, unlike with BCL-XL, BCL-W, and BCL-2 (Fig. 3c, d). Importantly, Co-IP assays demonstrated that N could interact with endogenous MCL-1 in A549-N cells (Fig. 3e), HEK293T-N cells (Fig. 3f), and THP-1-N differentiated macrophages (Fig. 3g). Additionally, although STS repressed MCL-1 protein level, N could still interact with MCL-1 in STS-treated cells (Fig. 3e-g). Meanwhile, Co-IP assay also proved that like WT-N protein, Dlta-N and Omic-N protein also interacted with MCL-1 protein (Supplementary Fig. 8a). Moreover, immunofluorescence showed that N and endogenous MCL-1 were co-localized in mitochondria (Fig. 3h). Collectively, SARS-CoV-2 N protein could interact with MCL-1 protein in the mitochondria of the cells.

It is known that MCL-1 contains four domains: PEST-like domain, BH1 domain, BH2 domain, and BH3 domain.^18^ Here, we determined the domain of MCL-1 involved in the interaction with N by constructing four MCL-1 mutants, ΔPEST, ΔBH3, ΔBH1, and ΔBH2, which lack each of the four domains, respectively (Supplementary Fig. 8b). Co-IP results showed that, like WT MCL-1, mutants ΔBH3, ΔBH1 and ΔBH2 could interact with N, except mutant ΔPEST (Supplementary Fig. 8c), suggesting that the PEST-like domain of MCL-1 was involved in the interaction with N. In addition, the sequences of N protein involved in the interaction with MCL-1 were identified by generating progressively truncated mutants of N protein, N1–N8 (Supplementary Fig. 8d). Co-IP results showed that N, N1(1–340), N5(90–420), N6(180–420) and N7(260–420) could interact with MCL-1, but N2(1–260), N3(1–180), N4(1–90) and N8(340–420) failed to interact with MCL-1 (Supplementary Fig. 8e), indicating the involvement of N domains containing 260aa-340aa in the interaction with MCL-1. Next, we used the Alphafold multimer software to predict the complex structure of N and MCL-1 (Supplementary Fig. 8f-j). The results showed that: PEST-like domain is mainly included loop structure (Supplementary Fig. 8f). The crystal structure of N protein has been reported, and it is dimer.^49^ The monomer structure of 260aa-340aa is shown in Supplementary Fig. 8g, and the dimer structure is shown in Supplementary Fig. 8h. The model of complex structure showed that 5 pairs of hydrogen bonds are formed in the complex, which indicated that the affinity coefficient of complex was high enough, and also proves the reliability of CO-IP results (Supplementary Fig. 8i). According to the simulation results, the complex may not affect the formation of the dimer of N protein, but may affect the conformation of the dimer (Supplementary Fig. 8j). Since the disordered loop region of MCL-1 PEST-like domain interacted with N protein, it is difficult to accurately predict the interaction site of the complex. Taken together, these results indicated that N protein domain 260aa–340aa interacted with MCL-1 protein PEST-like domain in the cells.

### N promotes MCL-1 K63-linked deubiquitination

**Fig. 4:**
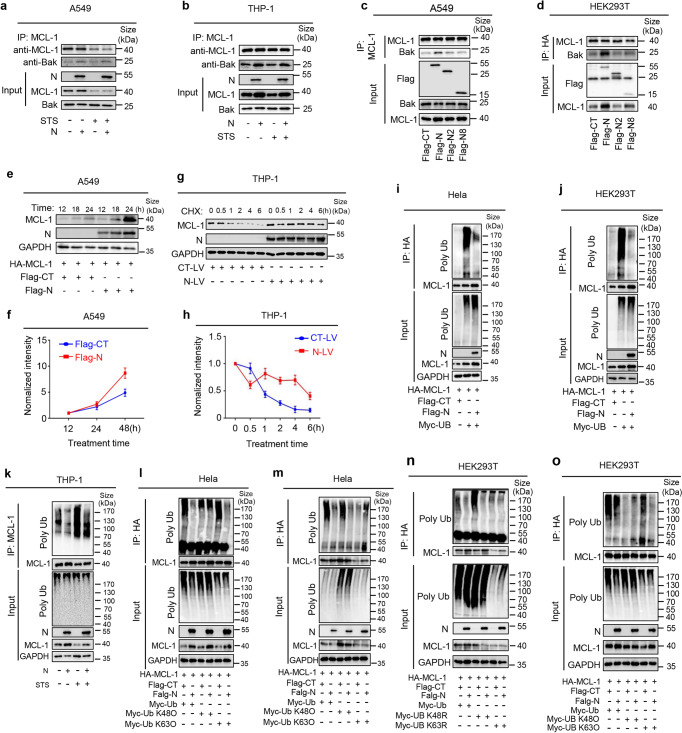
N promotes MCL-1 K63-linked deubiquitination. **a**, **b** A549 cells (**a**) or THP-1 cells (**b**) were stably infected with Lentivirus-CT or Lentivirus-N, THP-1 cells were differentiated into macrophages, then stimulated with 5 μM Staurosporine or DMSO for 4 h. Cell lysates were immunoprecipitated using an anti-MCL-1 antibody and analyzed using anti-Bax, anti-N and anti-MCL-1 antibody. Cell lysates (40 μg) were used as Input. **c**, **d** A549 cells (**c**) or HEK293T cells (**d**) were transfected with SARS-CoV-2-N protein and truncated mutants N protein (N2 and N8) for 24 h. Cell lysates were immunoprecipitated using an anti-MCL-1 antibody and analyzed using anti-Flag, anti-Bax and anti-MCL-1 antibodies. Cell lysates (40 μg) were used as Input. **e**, **f** A549 cells were respectively transfected with plasmid encoding MCL-1 (1 μg) plus Flag-ctrl (1 μg) or MCL-1 (1 μg) plus N (1 μg). The cells were collected at indicated time points (12 h, 18 h, and 24 h), and the indicated proteins in cell extract were analyzed by WB (**e**). Densitometry of MCL-1 immunoblot was analyzed by ImageJ (**f**). **g**, **h** THP-1 cells were stably infected with Lentivirus-CT or Lentivirus-N, differentiated into macrophages, then treated with protease inhibitor Cycloheximide (0.2 μM) for 30 min. The cells were collected at indicated time points (0, 0.5 h, 1 h, 2 h, 4 h, and 6 h), and the indicated proteins in cell extract were analyzed by WB (**g**). Densitometry of MCL-1 immunoblot was analyzed by ImageJ (h). **i**, **j** Hela cells (**i**) or HEK293T cells (**j**) were co-transfected with HA-MCL-1, Flag-N or Myc-Ubiquitin for 24 h. Cell lysates were immunoprecipitated using an anti-HA antibody and analyzed using anti-Myc, anti-Flag, anti-GAPDH, and anti-HA antibodies. Cell lysates (40 μg) were used as Input. **k** THP-1 cells were stably infected with Lentivirus-CT or Lentivirus-N, differentiated into macrophages, then stimulated with 5 μM Staurosporine or DMSO for 4 h. Cell lysates were immunoprecipitated using an anti-MCL-1 antibody and analyzed using anti- Ubiquitin, anti-N, anti-GAPDH and anti-MCL-1 antibody. Cell lysates (40 μg) were used as Input. **l**, **n** Hela cells (**l**) or HEK293T cells (**n**) were co-transfected with HA-MCL-1, Flag-N, Myc-Ubiquitin, Myc-Ubiquitin (K48O) or Myc-Ubiquitin (K63O) for 24 h. Cell lysates were immunoprecipitated using an anti-HA antibody and analyzed using anti-Myc, anti-Flag, anti-GAPDH and anti-HA antibodies. Cell lysates (40 μg) were used as Input. **m**, **o** Hela cells (**m**) or HEK293T (**o**) were co-transfected with HA-MCL-1, Flag-N, Myc-Ubiquitin, Myc-Ubiquitin (K48R) or Myc-Ubiquitin (K63R) for 24 h. Cell lysates were immunoprecipitated using an anti-HA antibody and analyzed using anti-Myc, anti-Flag, anti-GAPDH and anti-HA antibodies. Cell lysates (40 μg) were used as Input. Flag-CT indicates pcDNA3.1(+)-3×flag empty plasmid (**c**, **d**, **i**, **j**, **l**–**o**). Data are representative of three independent experiments, with one representative shown

As MCL-1 can suppress cell apoptosis by binding with pro-apoptosis protein Bak, here we explored the role of N in MCL-1/Bak interaction in cells treated with STS. The results showed that the interaction between endogenous MCL-1 with endogenous Bak was promoted by N in the cells (Fig. 4a, b). We observed that although STS repressed MCL-1, N could still facilitate endogenous MCL-1/endogenous Bak interaction (Fig. 4a, b). Notably, MCL-1/Bak interaction was enhanced by N in a dose-dependent manner (Supplementary Fig. 9a). Moreover, unlike WT N protein, truncated mutants N2 and N8 failed to promote MCL-1/Bak interaction (Fig. 4c, d), suggesting that N promoted MCL-1/Bak interaction.

Next, the mechanism via which N regulates MCL-1 was explored. Initially, the expression status of N and MCL-1 was examined. The results showed that the MCL-1 protein level was significantly enhanced by N protein in a time-dependent manner (Fig. 4e, f). THP-1 differentiated macrophages were then treated with Cycloheximide (CHX), an inhibitor for eukaryotic protein synthesis. Notably, the MCL-1 protein level was significantly decreased by CHX in a time-dependent manner in the absence of N protein, while it was slightly decreased by CHX in the presence of N protein (Fig. 4g, h), indicating that N could promote MCL-1 protein stability.

MCL-1 is regulated through transcriptional or post-transcriptional mechanisms. We observed that N protein had no influence on MCL-1 mRNA but promoted the MCL-1 protein, interacted with the MCL-1 protein and enhanced MCL-1 stability, suggesting that N regulated MCL-1 through a post-transcriptional mechanism. Then, we determined whether the ubiquitin-proteasome pathway was involved in the N-mediated regulation of MCL-1. Notably, in the presence of ubiquitin, the ubiquitination of MCL-1 was increased but significantly reduced by N (Fig. 4i, j). We also observed that the ubiquitination of endogenous MCL-1 was significantly reduced by N and promoted by STS, while STS-induced MCL-1 ubiquitination was significantly attenuated by N (Fig. 4k). Moreover, unlike WT N protein, the truncated N protein mutants N2 and N8) failed to promote MCL-1 deubiquitination (Supplementary Figs. 8, 9b, c). These results indicated that N protein could promote MCL-1 protein deubiquitination.

To determine the type of MCL-1 deubiquitination regulated by N, two ubiquitin mutants with only one lysine residue mutation (KR) and two ubiquitin mutants with only one single lysine residue (KO) were generated. The results indicated that MCL-1 ubiquitination was caused by UB, UB-K48R or UB-K63O, unaffected by UB-K63R or UB-K48O, and attenuated by N in the cells (Fig. 4l–o), demonstrating that MCL-1 ubiquitination was K63-linked and N could promote the removal of MCL-1 K63-linked ubiquitination. Taken together, these data showed that N regulated MCL-1 post-transcriptionally by promoting MCL-1 K63-linked deubiquitination.

### N-mediated MCL-1 deubiquitination requires USP15

**Fig. 5:**
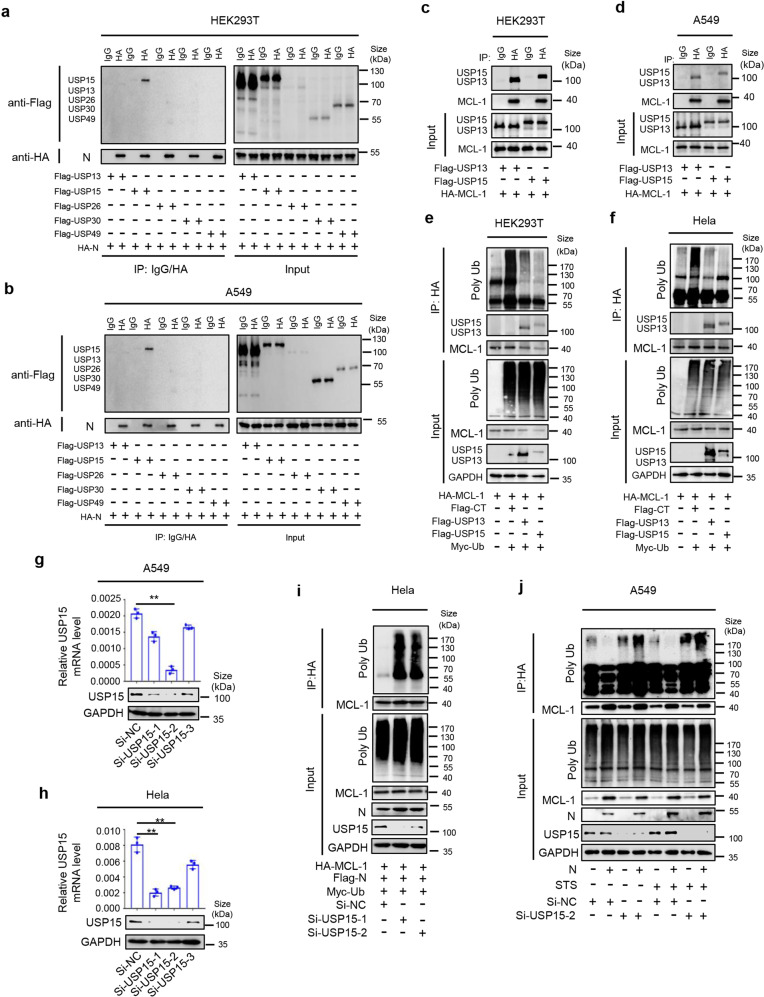
N-mediated MCL-1 deubiquitination requires USP15. **a**, **b** HEK293T cells (a) or A549 cells (b) were co-transfected with HA-N and Flag-USP13, Flag-USP15, Flag-USP26, Flag-USP30 or Flag-USP49 for 24 h. Cell lysates were immunoprecipitated using anti-HA or IgG antibodies and analyzed using anti-Flag and anti-HA antibodies. Cell lysates (40 μg) were used as Input. **c**, **d** HEK293T cells (**a**) or A549 cells (**b**) were co-transfected with HA-MCL-1 and Flag-USP13 or Flag-USP15 for 24 h. Cell lysates were immunoprecipitated using anti-HA or IgG antibodies and analyzed using anti-Flag and anti-HA antibodies. Cell lysates (40 μg) were used as Input. **e**, **f** HEK293T cells (**e**) or Hela cells (**f**) were co-transfected with HA-MCL-1, Flag-ctrl, Flag-USP13, Flag-USP15, or Myc-Ubiquitin for 24 h. Cell lysates were immunoprecipitated using an anti-HA antibody and analyzed using anti-Myc, anti-Flag, anti-GAPDH, and anti-HA antibodies. Cell lysates (40 μg) were used as Input. **g**, **h** A549 cells (**g**) or Hela cells (**h**) were transfected with si-NC, si-USP15-1, si-USP15-2 or si-USP15-3 (50 nM), respectively, for 48 h, and the mRNA levels of USP15 were quantified by qRT-PCR. Cell lysates were analyzed by immunoblotting. **i** Hela cells were firstly transfected with si-NC, si-USP15-1 or si-USP15-2 (50 nM) for 24 h, then co-transfected with HA-MCL-1, Flag-N or Myc-Ubiquitin for another 24 h. Cell lysates were immunoprecipitated using an anti-HA antibody and analyzed using anti-Myc, anti-Flag, anti-GAPDH, and anti-HA antibodies. Cell lysates (40 μg) were used as Input. **j** A549 cells were stably infected with Lentivirus-CT or Lentivirus-N by transfection with si-NC or si-USP15-2 (50 nM) for 48 h, then stimulated with 5 μM Staurosporine or DMSO for 4 h. Cell lysates were immunoprecipitated using an anti-MCL-1 antibody and analyzed using anti-Ubiquitin, anti-USP15, anti-N, anti-GAPDH, and anti-MCL-1 antibody. Cell lysates (40 μg) were used as Input. Flag-CT indicates pcDNA3.1(+)-3×flag empty plasmid (**e**, **f**). Data are representative of three independent experiments, with one representative shown. Error bars indicate SD of technical triplicates. Values are shown as mean ± SEM. *P ≤ 0.05, **P ≤ 0.01, ***P ≤ 0.001, two-tailed Student’s *t* test

The deubiquitinating enzymes (DUB) required for N-mediated MCL-1 deubiquitination were further explored. Initially, the roles of USP family members, including ubiquitin-specific peptidase 13 (USP13), USP15, USP26, USP30 and USP49, in N-mediated MCL-1 deubiquitination were evaluated. Co-IP assays showed that N could only interact with USP15, but failed to interact with USP13, USP26, USP30 and USP49 (Fig. 5a, b). A previous study reported that USP13 was potentially involved in the deubiquitination of MCL-1.^50^ Here, we showed that, like USP13, USP15 could interact with MCL-1 (Fig. 5c, d). The abundance of MCL-1 ubiquitination was increased in the presence of ubiquitin while significantly reduced by USP13 and USP15 in the cells (Fig. 5e, f).

Next, we investigated the role of USP15 in N-mediated MCL-1 deubiquitination. Three siRNAs (siUSP15-1, siUSP15-2 and siUSP15-3) specific targeting USP15 were generated. USP15 mRNA and USP15 protein were attenuated by siUSP15-2 while relatively unaffected by siUSP15-1 and siUSP15-3 (Fig. 5g). Similarly, USP15 mRNA and USP15 protein were notably reduced by siUSP15-1 and siUSP15-2, while relatively unchanged by siUSP15-3 (Fig. 5h). Further, MCL-1 ubiquitination was increased in the presence of N, ubiquitin, siUSP15-1 or siUSP15-2 (Fig. 5i). We also observed that in A549-N cells treated with or without STS, endogenous MCL-1 polyubiquitination was reduced in the absence of siUSP15-2; however, endogenous MCL-1 polyubiquitination was promoted in the presence of siUSP15-2 (Fig. 5j), suggesting that USP15 was required for N-mediated MCL-1 deubiquitination. In summary, the above results proved that USP15 is a ubiquitin-specific peptidase essential for the deubiquitination of MCL-1 protein mediated by SARS-CoV-2 N protein.

### N represses apoptosis to promote virus replication

**Fig. 6:**
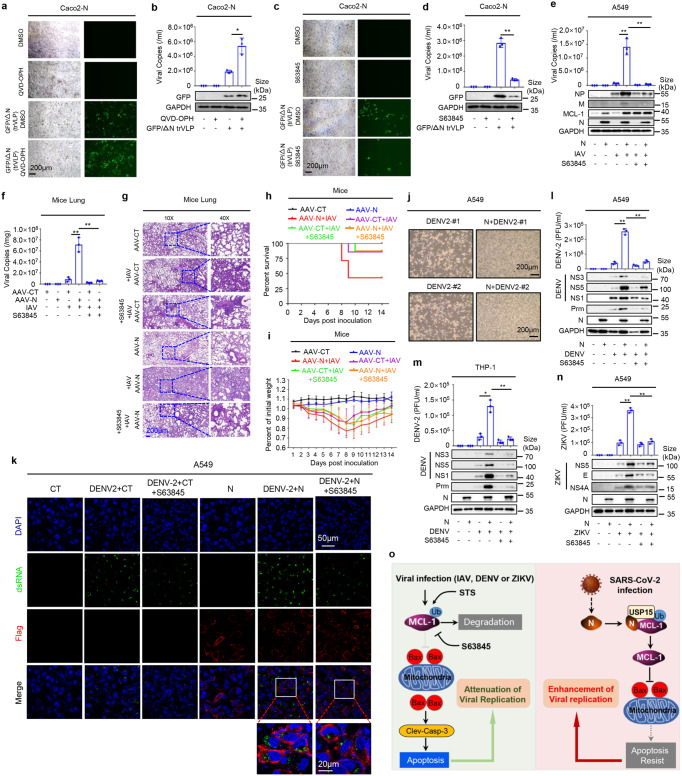
N represses apoptosis to promote virus replication. **a**, **b** Caco-2 cells were stably infected with Lentivirus-N, pre-treated with 50 μM apoptosis inhibitor QVD-OPH or DMSO for 4 h, then infected with SARS-CoV-2-trVLP (MOI = 0.5) for 48 h. GFP expression was observed in Caco2-N cells using microscopy (**a**). The viral copies in cell supernatant were absolute quantification of qRT-PCR (b, bottom). Cell lysates were analyzed by immunoblotting (**b**, lower). **c**, **d** Caco-2 cells were stably infected with Lentivirus-N, pre-treated with 3 μM MCL-1 inhibitor S63845 or DMSO for 4 h, then infected with SARS-CoV-2-trVLP (MOI = 0.5) for 48 h. GFP expression was observed in Caco2-N cells using microscopy (**c**). The viral copies in cell supernatant were absolute quantification of qRT-PCR (**d**, bottom). Cell lysates were analyzed by immunoblotting (**d**, lower). **e** A549 cells were stably infected with Lentivirus-CT or Lentivirus-N, pre-treated with 3 μM MCL-1 inhibitor S63845 or DMSO for 4 h, then infected with influenza virus (PR8 strain, MOI = 0.1) for 48 h. The viral copies in cell supernatant were absolute quantification by qRT-PCR (**e**, lower). **f**–**i** C57BL/6 genetic background mice were injected with 300 μl containing 3 × 10^11^ vg of AAV-Lung-EGFP (*n* = 20) or AAV-Lung-N (*n* = 2 0) in their tail vein, and after three weeks, pre-treated with MCL-1 specific inhibitor S64845 (12.5 mg/kg) by intraperitoneal injection for 90 min and then infected with influenza virus (PR8 strain, 1LD50) by intranasal injection, followed by repeated treatment with S64845 (12.5 mg/kg) on the fourth day after influenza virus infection (*n* = 8), or the same volume solvent (50% PEG300 plus 50% PBS) as a control group (*n* = 8). Another untreated AAV-Lung-EGFP or AAV-Lung-N infected mice were prepared as the blank group (*n* = 4). Six days after infection, two mice were euthanized, and their lung tissues were collected. The viral copies in lung tissues were absolute quantification of qRT-PCR (**f**). Histopathology analysis of the mice’s lungs (**g**). Scale bar, 200 μm (10×) or 50 μm (40×). The mice’s survival rates (**h**) and weight changes (**i**) were evaluated every day post-treatment. **j** A549 cells were stably infected with Lentivirus-CT or Lentivirus-N, then infected with Dengue virus (NGC strain, MOI = 0.5) for 48 h, Cytopathic effect (CPE) was observed in above cells using microscopy. **k** A549 cells were stably infected with Lentivirus-CT or Lentivirus-N, pre-treated with 3 μM MCL-1 inhibitor S63845 for 4 h. then infected with Dengue virus (NGC strain, MOI = 0.5) for another 48 h. The sub-cellular locations of virus-dsRNA (green), Flag-tagged N (red), and nucleus marker DAPI (blue) were visual with confocal microscopy. Scale bar is 50 μm. **l**–**n** A549 cells (**l**, n)or THP1- cells (**m**) were stably infected with Lentivirus-CT or Lentivirus-N, THP-1 cells were differentiated into macrophages. Pre-treated with 3 μM MCL-1 inhibitor S63845 for 4 h. then infected with Dengue virus (NGC strain, MOI = 0.5) (**l**, **m**) or ZIKV virus (PRVABC.59 strain, MOI = 0.5) (**n**) for another 48 h. Cell supernatant was analyzed by plaque (upper) and cell lysates by immunoblotting (lower). **o** A proposed model in which SARS-CoV-2 N protein suppresses apoptosis to promote virus replication through regulating MCL-1 protein. GFP/ΔN trVLP means SARS-CoV-2-GFP/ΔN trVLP (**a**–**d**). Data are representative of two independent experiments, with one representative shown. Error bars indicate the SD of each serum sample; *P* ≤ 0.05 (*), *P* ≤ 0.01 (**), *P* ≤ 0.001 (***), and two-tailed Student’s *t* test (**b**, **d**, **e**, **f**, **l**, **m**, and **n**). One-way ANOV analysis (**h**)

Previous studies reported that N protein promoted virus replication by impairing stress granule formation or regulating the IFN pathway.^30,31^ Here, we identified the impact of cell apoptosis on SARS-CoV-2 replication. We pre-treated Caco2-N cells with QVD-OPH (an inhibitor of apoptosis), then infected with SARS-CoV-2-trVLP. The results showed that SARS-CoV-2-trVLP replication (as indicated by the GFP signal) (Fig. 6a), SARS-CoV-2-trVLP copies in cell supernatants (Fig. 6b, upper) and GFP protein in cell lysates (Fig. 6b, low) were promoted by QVD-OPH. In contrast, SARS-CoV-2-trVLP replication (Fig. 6c), viral copies (Fig. 6d, upper) and GFP production (Fig. 6d, low) were reduced by S63845 in Caco-2-N cells pre-treated with S63845 and infected with SARS-CoV-2-trVLP. These results suggested that the inhibition of apoptosis promoted SARS-CoV-2-trVLP replication, while induction of apoptosis repressed viral replication.

The roles of N protein and cell apoptosis in the replications of other viruses were assessed. Previous research has found that IAV infection promoted SARS-CoV-2 infection.^33^ Here, we showed that in A549-N cells pre-treated with S63845 and infected with IAV, IAV viral copies in cell supernatants and protein productions were promoted by N but reduced by S63845 (Fig. 6e). In A549-CT cells and A549-N cells transfected with siNC, siMCL-1-1 or siMCL-1-2 and infected with IAV, IAV NP and M proteins were increased by N, but such inductions were repressed by siMCL-1-1 or siMCL-1-2 (Supplementary Fig. 10a). Further, IAV NP was enhanced by N but relatively unaffected by N2 and N8 mutants (Supplementary Fig. 10b). These results indicated that N could promote IAV replication by regulating cell apoptosis and MCL-1.

Next, the effects of N and cell apoptosis on IAV infection were explored in AAV-Lung-N C57BL/6 mice. AAV-Lung-CT C57BL/6 mice and AAV-Lung-N C57BL/6 mice were infected with IAV and pre-treated with S63845. We found that in the lungs of IAV-infected mice, IAV viral copies were enhanced by AAV-N but repressed by S63845 (Fig. 6f). Hematoxylin & Eosin (H&E) staining showed that in AAV-Lung-N C57BL/6 mice lung infected with IAV, tissue injuries were very high. However, such pathological changes were suppressed by S63845 (Fig. 6g). Importantly, mice survival rates (Fig. 6h) and mice weights (Fig. 6i) were attenuated upon IAV infection and further reduced by AAV-N, while such repressions were recovered by S63845 (Fig. 6h, i). Collectively, N protein could enhance IAV replication by regulating cell apoptosis and MCL-1 in mice.

In addition, the role of N protein in Dengue virus 2 (DENV-2) replication was assessed. We observed cytopathic effects (CPEs) in A549-CT cells infected with DENV-2 and reduced CPEs in A549-N cells infected with DENV-2 (Fig. 6j), indicating that N could repress DENV-2-induced apoptosis. Confocal microscope analyses revealed that in A549-N cells infected with DENV-2, the viral dsRNA (in green) was promoted but could be reduced with S63845 (Fig. 6k). Next, cells were pre-treated with S63845, then infected with DENV-2. The results showed that in the cells infected with DENV-2, DENV-2 viral titers as well as NS3, NS5, NS1 and Prm protein levels were increased, but such inductions were reduced by S63845 (Fig. 6l, m). Notably, DENV-2 viral titers, along with NS1 and Prm proteins, were promoted by N but not by truncated proteins N2 and N8 (Supplementary Fig. 10c).

Lastly, the effects of N protein on Zika virus (ZIKV) replication were determined. The results showed that in cells pre-treated with S63845 and infected with ZIKV, ZIKV viral titers and ZIKV NS5, E and NS4A protein levels were up-regulated by N while repressed by S63845 (Fig. 6n). Notably, ZIKV viral titers and ZIKV NS5 and NS3 protein levels were promoted by N in A549-N cells, but not by truncated protein N2 in A549-N2 cells or truncated protein N8 in A549-N8 cells (Supplementary Fig. 10d). These results indicated that N protein enhanced ZIKV replication by promoting MCL-1 and repressing cell apoptosis.

Taken together, SARS-CoV-2 N protein could inhibit cell apoptosis and promote the replications of viruses, including SARS-CoV-2, IAV, DENV-2 and ZIKV, by regulating the MCL-1 protein (Fig. 6o).

## Discussion

As important physiological processes of the body, the cell death pathways play crucial roles in maintaining the homeostasis of organisms and pathogenic microbes.^16,44^ A previous study reported that SARS-CoV-2 N facilitated the cleavage and activation of Casp-1.^27^ It indicated that N promoted pyroptosis, as Casp-1 activation is a key step in the pyroptotic pathway.^46^ N also facilitated the cleavage of another key protein Gasdermin D (GSDMD) in the pyroptotic pathway,^46^ suggesting that N induced pyroptosis through the NLRP3 inflammasome. In this study, we showed that N repressed cell apoptosis by attenuating Casp-3 cleavage, a marker of the apoptosis pathway,^17^ by regulating MCL-1, an important anti-apoptotic protein.^18^ The intrinsic apoptosis pathway includes mitochondria and BCL-2 family members.^16^ BCL-2 family proteins are the main regulators of apoptotic processes and consist of anti-apoptotic members (BCL-2, BCL-X, BCL-XL and MCL-1) and pro-apoptotic members (Bax, Bak, Bad, Bid and Bim).^17^ Here, we found that N regulated MCL-1 function to repress cell apoptosis, and that the inhibitor of MCL-1 (S63845) or knock-down of MCL-1 eliminated the anti-apoptotic effects of N. We speculate that the cell apoptosis repression may be important for maintaining the normal structure and function of respiratory tract in asymptomatic infected individuals. This may explain the high frequency of asymptomatic SARS-CoV-2 infection.

MCL-1 comprises the PEST-like domain on N-terminal and multiple BH domains on the C-terminal.^18^ The PEST-like domain has characteristic sequences found in proteins with rapid turnover, which are thought to be the reasons for the short half-life of MCL-1.^18^ We showed that 260aa–340aa of N could stabilize MCL-1 by interacting with the PEST-like domain. Although we have made the complex structural prediction by Alphafold multimer software, since the disordered loop region of MCL-1 PEST-like domain interacted with N protein, it was difficult to accurately predict the interaction site which needs to be further explored in the future. MCL-1 BH domains could neutralize pro-apoptotic proteins by binding to the proteins.^18^ We proved that N promoted MCL-1/Bak interaction, but mutants N2 and N8 of N failed to interact with MCL-1 and had no effects on MCL-1/Bak interaction. MCL-1 was regulated at the transcriptional, translational and post-translational levels. We demonstrated that N did not influence MCL-1 transcription, while it enhanced MCL-1 protein level by protecting MCL-1 from degradation by enhancing K63-linked deubiquitination and recruiting deubiquitinating enzyme USP15. As protein ubiquitylation is very complicated process, and is catalyzed by different enzymatic reactions including E1, E2, E3 and ubiquitin. It has been shown that in mammalian cells, there are 2 types of E1s, 38 of E2s, more than 600 of E3s,and 106 DUBs. It is probably that N protein enhances MCL-1 stability by regulating an unknown E3 ubiquitin ligase, which requires further explored in the future. Therefore, we uncovered a distinct mechanism by which N regulated MCL-1 to repress cell apoptosis.

As IAV and SARS-CoV-2 infect the same respiratory tract, the co-infection of the two viruses was clinically detected. The results showed that IAV infection enhanced SARS-CoV-2 infection by promoting ACE2 expression.^33,51,52^ However, the effects of SARS-CoV-2 on IAV infection remain unknown. Here, we showed that N promoted IAV replication but was repressed by MCL-1 knock-down or S63845 administration. Notably, N promoted the death of IAV-infected mice, while S63845 delayed the death time and improved the survival rate of the mice. In tropical and subtropical regions, there are also clinical reports of SARS-CoV-2 co-infecting with DENV^53^ and ZIKV.^54^ We showed that N enhanced the replication of DENV and ZIKV, while S63845, the inhibitor of MCL-1, repressed DENV and ZIKV replication. Compared with N protein, the mutants N2 and N8 of N fail to interact with MCL-1 and could not promote viral replication. These results demonstrated that N promoted virus replication by repressing MCL-1 and cell apoptosis.

N functionally encapsulates the viral RNA and plays an vital role in viral replication.^26^ N directly interacts with NLRP3 to promote the NLRP3 inflammasome assembly and activation and induces cytokine storm, thereby causing lung injury.^27^ N also induces acute kidney injury via a Smad3-dependent mechanism^28^ and represses RIG-I mediated IFN-β production.^29^ Further, N promotes viral replication by impairing stress granule formation^30^ and suppresses antiviral immune response by regulating MAVS activity.^31^ In this study, we identified a new function of N, whereby it could repress cell apoptosis and promote virus replication. Our data also suggest that N may play a role in the co-infection of SARS-CoV-2 with other viruses, although such function should be validated in future studies.

In summary, this study revealed a unique anti-apoptotic mechanism by which SARS-CoV-2 inhibited cell apoptosis to promote virus replication through its N protein. N interacted with the anti-apoptotic protein MCL-1 to facilitate the interaction of MCL-1 with the pro-apoptotic protein Bak. In addition, N interacted with MCL-1 to promote MCL-1 K63-linked deubiquitination and protected it from degradation by recruiting the deubiquitinating enzyme USP15. Furthermore, N also facilitated the replication of RNA viruses, including IAV, DENV and ZIKV, by repressing cell apoptosis, as inhibitor S63845 treatment or MCL-1 knock-down eliminated N antiviral effects. Based on these findings, we anticipate that MCL-1 inhibition and apoptosis could be potential therapeutic strategies for preventing and treating viral co-infections.

## Materials and methods

### Animal study

Wild-type C57BL/6 mice were purchased from Guangdong Medical Laboratory Animal Center, Guangzhou, China. Mice were cultivated and maintained under specific pathogen-free conditions at Jinan University. Four weeks old mice were infected with 300 μl of AAV-Lung-EGFP-N (5 × 10^11^ vg) or AAV-Lung-EGFP (purchased from OBiO Technology, Shanghai, China) by the tail vein injection. After three weeks, the mice were pre-treated with the MCL-1 specific inhibitor-S63845 (12.5 mg/kg) by intraperitoneal injection, then infected with influenza virus (1LD50) by intranasal injection. The mice were sacrificed, and their lung tissues were collected for histoimmunofluorescence or histopathology analysis.

### Establishment of human lung organoids

Human Lung organoids were established from lung tissues of donors undergoing surgery for non-small-cell lung cancer. Briefly, distal normal tissues (at least 5 cm away from the tumor) were collected post-surgery from the resected specimen and processed as previously described.^55^ The tissues were partially digested, filtrated and centrifuged, then resuspended in Matrigel and cultured with CMGF+ media in a 37 °C incubator (regular 5% CO_2_). The medium was changed every 3-4 days, and the organoids were passaged every 14 days for 2 months before the experiment.

### Ethics statement

All included lung tissues of donors gave their oral and written informed consent. The study was conducted according to the principles of the Declaration of Helsinki and approved by the Ethics committee of The First People’s Hospital of Foshan in accordance with its guidelines for the protection of human subjects (approval number: FSYYY-EC-SOP-008-02.0-A09).

All animal studies were performed in accordance with the principles described by the Animal Welfare Act and the National Institutes of Health Guidelines for the care and use of laboratory animals in biomedical research. The experimental protocols and all procedures involving mice were approved by the Institute of Laboratory Animal Science, Jinan University (approval number: 20220302-25). All the mice were sacrificed by euthanasia.

### The system of SARS-CoV-2 virus-like particle (trVLP)

The full-length SARS-CoV-2 GFP/ΔN cDNA was kindly provided by Dr. Qiang Ding, Tsinghua University. Then, the viral RNA transcript transcribed in vitro was electroporation into Caco-2 cells stably expressing SARS-Cov-2 N gene (i.e. Caco-2-N cells). Within 48 h, GFP fluorescence was observed, and after 96 h, cytopathic effect (CPEs) was observed, indicating the production and reproduction of recombinant SARS-CoV-2 virus like particles (trVLP). Cell culture supernatants were obtained and centrifuged at 1000 rcf for 10 min to remove cell fragments. Then, using 0.22 μM filter membrane filter them. Divide all viruses into tubes and freeze at −80 °C.

### Cell culture

We purchased Human pulmonary epithelial cell (A549), human cervical carcinoma cell (Hela), human lung microvascular endothelial cells (HULEC), African green monkey kidney cell (Vero), human embryonic kidney cell lines (HEK-293T) and human monocytic cell lines (THP-1) from the American Type Culture Collection (ATCC). The above cells were cultured in DMEM (HEK293T and A549), MEM (Vero), ECM (HULEC), and RPMI1640 (THP-1) medium, respectively. All the above media required supplementation with 10% fetal bovine serum, 100U/ml penicillin, and 100% μg/ml streptomycin, culture temperature at 37 °C, culture condition with 5% CO_2_. THP-1 cells were differentiated into macrophages by stimulating them with phorbol-12 myristic acid-13 acetate (PMA) for 12 h. Then, the cells were cultured in fresh medium for another 24 h.

### Virus

All experiments used influenza virus (IAV, PR8 strain), Dengue virus (DENV-2, NGC strain) and Zika virus (ZIKV, PRV08 strain), which were stored by our lab. To generate large stocks of IAV, eight-day-old SPF embryos were infected with IAV at MOI of 0.1 for 4-7 days, then supernatants of embryos including IAV were harvested and centrifuged at 1000 rpm for 10 min and filtrated by 0.44 μm filter membrane. The virus was aliquoted into tubes for freezing at −80 °C. To generate large stocks of DENV-2 or ZIKV, C6/36 cells were infected with DENV-2 or ZIKV at MOI of 0.5 for 2 h, then washed the unbound viruses. The infected cells were incubated sequentially in a fresh medium containing 2% FBS for another 7 days. The supernatants were harvested and centrifuged at 4000 rpm for 10 min to remove cellular debris. Then, they were filtrated by using a 0.22 μm filter membrane. All virus was aliquoted into tubes and freezing them at −80 °C. Virus tiles were determined by plaque assay using Vero cells.

### Reagents and antibodies

We purchased Phorbol-12-myristate-13-acetate (PMA), Lipofectamine 2000, and Trizol from Sigma-Aldrich, Invitrogen, Ambion company, respectively. Staurosporine (S1421), Rapamycin (S1039) and Erastin (S7242) were purchased from Selleck. S63845 (HY-100741) was purchased from MedChemExpress. FITC Annexin V Apoptosis Detection Kit I (556547) was purchased from BD Biosciences. Cell Counting Kit-8 (C0037) was purchased from Beyotime.

Anti-Caspase-8 (D35G2), anti-Caspase-3 (9962), Anti-GSDMD (E3E3P), anti-Bim (C34C5), Anti-Bak (D4E4), anti-BCL-XL (54H6), Anti-BCL-W (31H4), anti-MCL-1 (D2W9E), anti-UB (P4D1), anti-USP15 (D1K6S) and anti-Bax (D2E11) antibodies were purchased from Cell Signaling Technology. Anti-SARS-CoV-2-N protein (A20021), anti-NOX1 (A8527), anti-GPX4 (A1933), anti-LC3 (A19665), anti-P62 (A19700), anti-Myc (AE009) and anti-β-actin (AC026) antibody were purchased from ABclonal Technology. We purchased Flag (F3165), HA (H6908) and GAPDH (G8759) antibodies from Sigma. Anti-DENV-NS3 (GTX124252), anti-DENV-NS5 (GTX103350), anti-DENV-Prm (GTX128092), Anti-ZIKV-NS5 (GTX133312), anti-ZIKV-E (GTX133314), Anti-ZIKV-NS4A (GTX133704), anti-ZIKV-NS3 (GTX133309) antibodies and anti-IAV-NP (GTX125989) were purchased from GeneTex. Anti-DENV-NS1 (SQab1501) was purchased from Arigo Biolaboratories. Anti-IAV-M (ab22396) was purchased from Abcam. The immunoglobulin G (IgG) of control mice and rabbit used in the CO-IP experiment was purchased from Invitrogen, while the IgG of Dylight 649, cy3, and FITC labeled mice and rabbits in the immunofluorescence secondary antibody were purchased from Abbkine.

### RNA extract and qRT-PCR

According to the manufacturer’s instructions, using Trizol reagent (Invitrogen, Carlsbad, CA) to extract total RNA. We used Roche LC480 for relative quantitative analysis of target genes by Real-time quantitative reverse transcriptase-PCR (qRT-PCR). To quantify viral copies in cell supernatant or lung tissues, ten-fold serial dilutions of plasmid containing viral protein (1 × 10^1^ to 1 × 10^8^ template copies per reaction) were analyzed by qRT-PCR, and total viral copies were calculated from Ct values using the resulting standard curves. We used Primer-blast, NCBI (www.ncbi.nlm.nih.gov) to design Real-time PCR primers and list the sequences in in Supplementary Table S1.

### Clone construction

Plasmids pcDNA3.1(+)-3×flag-N/M/E/3a/6/7a/8/10 and the truncated forms of SARS-CoV-2-N (N1–N8), plasmids pCAGG-HA-N/MCL-1/BCL-2/BCL-XL/BCL-W and the truncated forms of MCL-1 (pcDNA3.1(+)-3×flag-ΔPEST, ΔBH1, ΔBH2, and ΔBH3), plasmids pCMV-MYC-UB/K48R/K63R/K48O/K63O were stored in our own laboratory.

### Stable cell lines construction

In our previous research, we have successfully constructed stably expressed N-lentivirus and CT-Lentivirus THP-1 cells. The procedure was as follows: Firstly, the plasmid pLenti-3×flag-N or control empty vector were co-transfected into HEK293T cells using Lipo2000 with packaging plasmids psPAX2 and pMD2G. Secondly, replace the old medium with fresh medium containing 2% FBS 12 h after transfection. Then, 36 h and 60 h after transfection, collected the cell supernatants containing lentivirus, and used 0.45 μm filter filtration. THP-1, HEK293T and A549 cells were infected with the collected lentivirus for 24 h by adding polystyrene (Sigma, TR-1003). Finally, positive cells were screened using purinomycin (Sigma, P8833) for another 4–7 days.

### Lentiviral infection of human lung organoids

The medium was removed and replaced with an ice-cold cell recovery solution for lentiviral infection. The Organoid-Matrigel drops were broken mechanically with pipette tips and incubated at 4 °C with shaking to further dissolve the Matrigel. After 20–30 min, the medium was centrifuged (400*g*, 5 min), and the supernatant was removed. The organoids were then cultured with the lentiviral solution (lentiviral supernatant mix with an equal volume of CMGF+ media and supplemented with 0.1% polybrene) in a low attachment 24 well plate for 72 h. The infected organoids were then used for the following experiments.

### Western blot

The cells were collected at indicated time and washed twice with cold PBS, then lysed in lysis buffer. Protein concentration was detected with the Bradford assay (Bio-Rad, Richmond, CA). Cell lysates (50 μg) were electrophoresed on 8−12% SDS PAGE and transferred to nitrocellulose membranes (Amersham, Piscataway, NJ). Nonspecific bands of NC membranes were blocked using 5% skim milk for 2 h. NC membranes were washed three times with PBS containing 0.1% Tween (PBST) and incubated with indicated antibodies. Protein bands were visualized using a Bio-Rad Image Analyzer (Serial No. 733BR3722).

### Co-immunoprecipitation assay

Co-transfected HEK293T and A549 cells with indicated plasmids for 24 h, and stimulated HEK293T/A549/THP-1-N stable cells with Staurosporine. Collected and lysed the above cells. Rotated the lysates at 4 °C for 30 min and then centrifuged at 13,000*g* for 10 min to remove excess cell fragments. Collected cell supernatants, take out a portion of the supernatants as input, incubated the remaining lysates with designated antibodies at 4 °C overnight, then mix with protein G Sepharose beads (GE Healthcare, Milwaukee, WI, USA) at 4 °C for 2 h. The immunoprecipitates were washed 4–6 times with eluent, added with protein loading buffer, boiled in boiling water for 10 min, and analyzed by Western blotting.

### Immunofluorescence assay

The stably expressed N-Lentivirus A549 cells were grown on confocal dishes for 24 h. Next, stablely expressed N-Lentivirus and CT-Lentivirus A549 cells grown on confocal dishes were pretreated with S63845, and infected with DENV (MOI = 0.5) for 48 h. Collected the above cells, removed the culture medium, and fixed the cells with 4% paraformaldehyde for 30 min, then permeabilized with PBS containing 0.1% TritonX-100 for 5 min. Finally, closed with PBS containing 5% BSA for 1 h. Incubated the cells with the corresponding primary antibody overnight, and then staining with Cy3, FITC, and 649 coupled IgG secondary antibodies for 30 min. The nucleus were stained with DAPI for 5 min, washed with cold PBS 3 times, and observed under confocal fluorescence microscopy (Leica, TCS, SP8).

### Cell counting Kit-8 (CCK8) assay

The stably expressed N-Lentivirus and CT-Lentivirus A549 cells and THP-1 cells were subsequently differentiated into macrophages. The cells were grown in a 96-well plate, then stimulated with Staurosporine. The changes in cell proliferation were measured with a CCK8 Kit following the manufacturer’s instructions. Briefly, 2000 cells in 100 µl were added to each well, to which the 10 µl CCK-8 solution was added at indicated time (0, 6, 12, 24, and 48 h), incubated at 37 °C in a 5% CO_2_ incubator for 1 h, and measured their absorbance were measured at 450 nm.

### Flow cytometry

The stable expressing N-Lentivirus and CT-Lentivirus A549 cells and THP-1 cells were subsequently differentiated into macrophages. The cells were pre-treated with S63845 for 4 h, then stimulated with Staurosporine. The cell apoptosis level was measured with a FITC Annexin V Apoptosis Detection Kit I according to the manufacturer’s instructions. Briefly, the collected cells were washed twice with cold PBS, then resuspended in 1× Binding Buffer at a concentration of 1 × 10^6^ cells/ml. They were then transferred into 100 µl of the solution (1 × 10^5^ cells) to a 5 ml culture tube, to which 5 µl of FITC Annexin V and 5 µl PI were added, gently vortexed and incubated for 15 min at 25 °C in the dark. Lastly, 400 µl of 1 × Binding Buffer was added to each tube, which was then analyzed by flow cytometry within 1 h.

### Statistical analyses

All experiments were repeated at least three times. The *t* test was used to compare two groups and one-way ANOVA for multiple groups (GraphPad Prism7). The data were defined statistically significant when *P* ≤ 0.05 (*), *P* ≤ 0.01 (**) and *P* ≤ 0.001 (***).

## Supplementary information

supplementary manuscript-docx

## Data Availability

The data used in the current study are available from the corresponding authors upon reasonable request.
