# Supplementary material for: SARS-CoV-2 N protein enhances the anti-apoptotic activity of MCL-1 to promote viral replication
Source: Signal Transduct Target Ther. 2023 May 9;8:194. doi: 10.1038/s41392-023-01459-8 (PMC10169150; doi:10.1038/s41392-023-01459-8)
Supplement: Supplementary file 1 — supplementary manuscript-docx [file 41392_2023_1459_MOESM1_ESM.docx]

Supplementary Materials for

SARS-CoV-2 N protein enhances the anti-apoptotic activity of MCL-1 to promote viral replication

Pan Pan^1,2,3,# *^, Weiwei Ge^4,#^, Zhiwei Lei^3,#^, Wei Luo ^5,#^, Yuqing Liu^3^, Zhanwen Guan^5^, Lumiao Chen^1^, Zhenyang Yu^4^, Miaomiao Shen^4^, Dingwen Hu^4^, Qi Xiang^4^, Wenbiao Wang^3^, Pin Wan^2^, Mingfu Tian^1^, Yang Yu^3^, Zhen Luo^3^, Xulin Chen^3^, Heng Xiao^3^, Qiwei Zhang^3^, Xujing Liang^1^, Xin Chen^3,*^, Yongkui Li^3,*^, and Jianguo Wu^1,2,3,4, &^

**Correspondence to:** Pan Pan, E-mail: [panpan@jnu.edu.cn](mailto:panpan@jnu.edu.cn); Xin Chen, E-mail: chenx@jnu.edu.cn; Yongkui Li, E-mail: [lyk070@jnu.edu.cn](mailto:lyk070@jnu.edu.cn)

**This PDF file includes:**

Figures. S1 to S10

Tables S1

**Figure. S1**

**
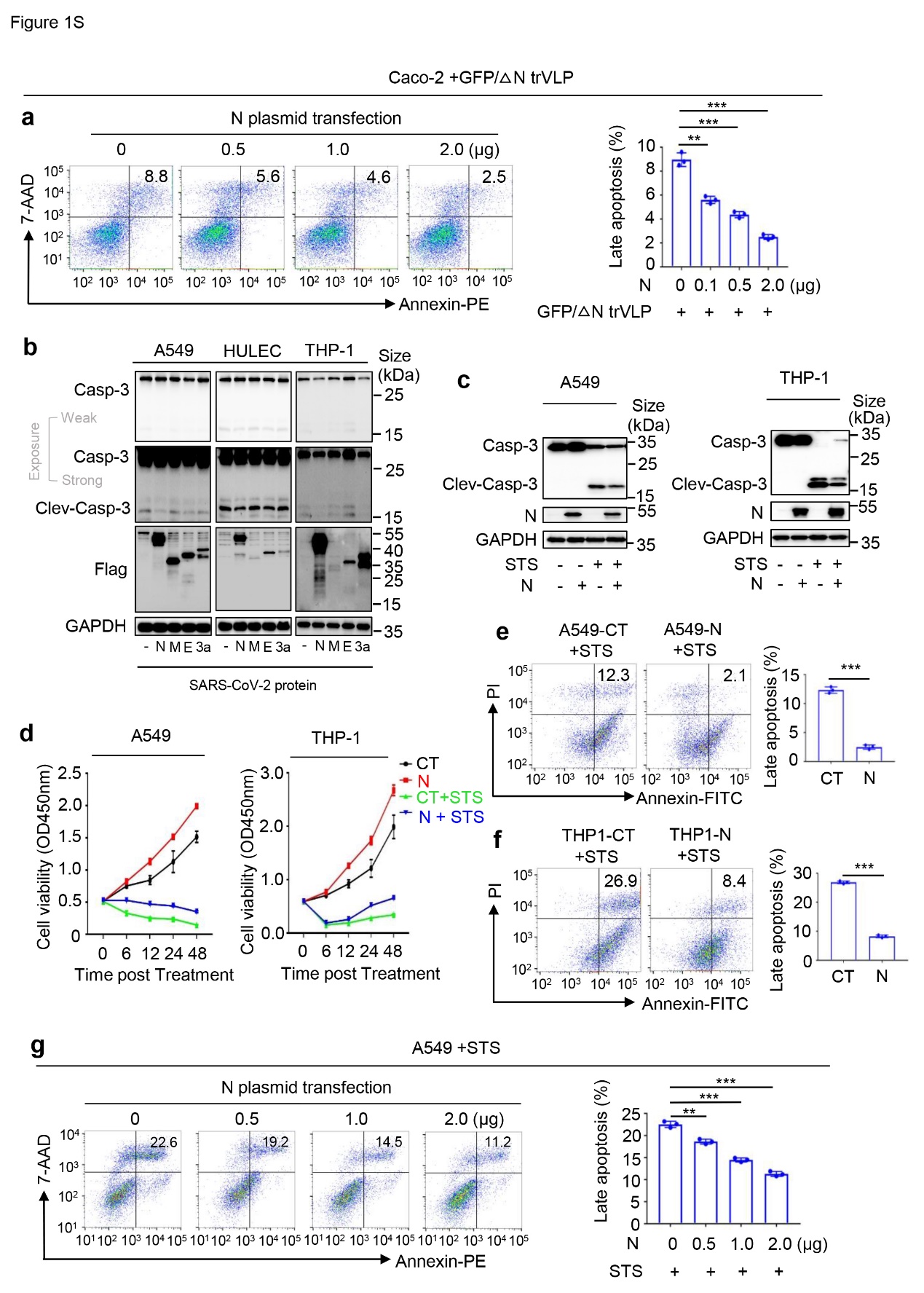
**

**Figure S1. SARS-CoV-2 N protein inhibits cell apoptosis. (a)** Caco-2 cells were transfected with different concentrations of plasmids encoding N for 24 h, and then infected with transcription and replication-competent SARS-CoV-2 virus-like-particles (SARS-CoV-2-trVLP) (MOI=0.5) for another 24h. The cell apoptosis level was analyzed by Flow cytometry (a, left) and the late apoptosis percent (7-AAD^+^ and Annexin^+^) were statistics (a, right). **(b)** A549 cells (b, left), HULEC cells (b, middle) or differentiated THP-1 cells (b, right) were transfected with plasmids encoding N, M, E, 3a for 48 h, and then stimulated with 5 μM Staurosporine or DMSO for 4 h. Cell lysates were analyzed by immunoblotting. **(c, d)** A549 and THP-1 cells were stably infected with Lentivirus-CT or Lentivirus-N, THP-1 cells were differentiated into macrophages, and then stimulated with 5 μM Staurosporine or DMSO for 4 h. Cell lysates were analyzed by immunoblotting (c). CCK8 analysis shown cell viability at different time points (d). **(e, f)** A549 cells were stably infected with Lentivirus-CT or Lentivirus-N (e), THP-1 cells were stably infected with Lentivirus-CT or Lentivirus-N, differentiated into macrophages (f), and then stimulated with 5 μM Staurosporine or DMSO for 4 h. The cell apoptosis level was detected by Flow cytometry. The late apoptosis percent (PI^+^ and Annexin^+^) were statistics. **(g)** A549 cells were transfected with different concentrations of plasmids encoding N for 24 h, and then stimulated with 5 μM Staurosporine for 4 h. The cell apoptosis level was detected by Flow cytometry. The late apoptosis percent (7-AAD^+^ and Annexin^+^) were statistics. CT means CT-lentivirus (d, e, f). Data are representative of three independent experiments and one representative is shown. Error bars indicate SD of technical triplicates. Values are mean± SEM. *P≤0.05, **P≤0.01, ***P≤0.001, two-tailed Student’s t-test.

**Figure. S2**


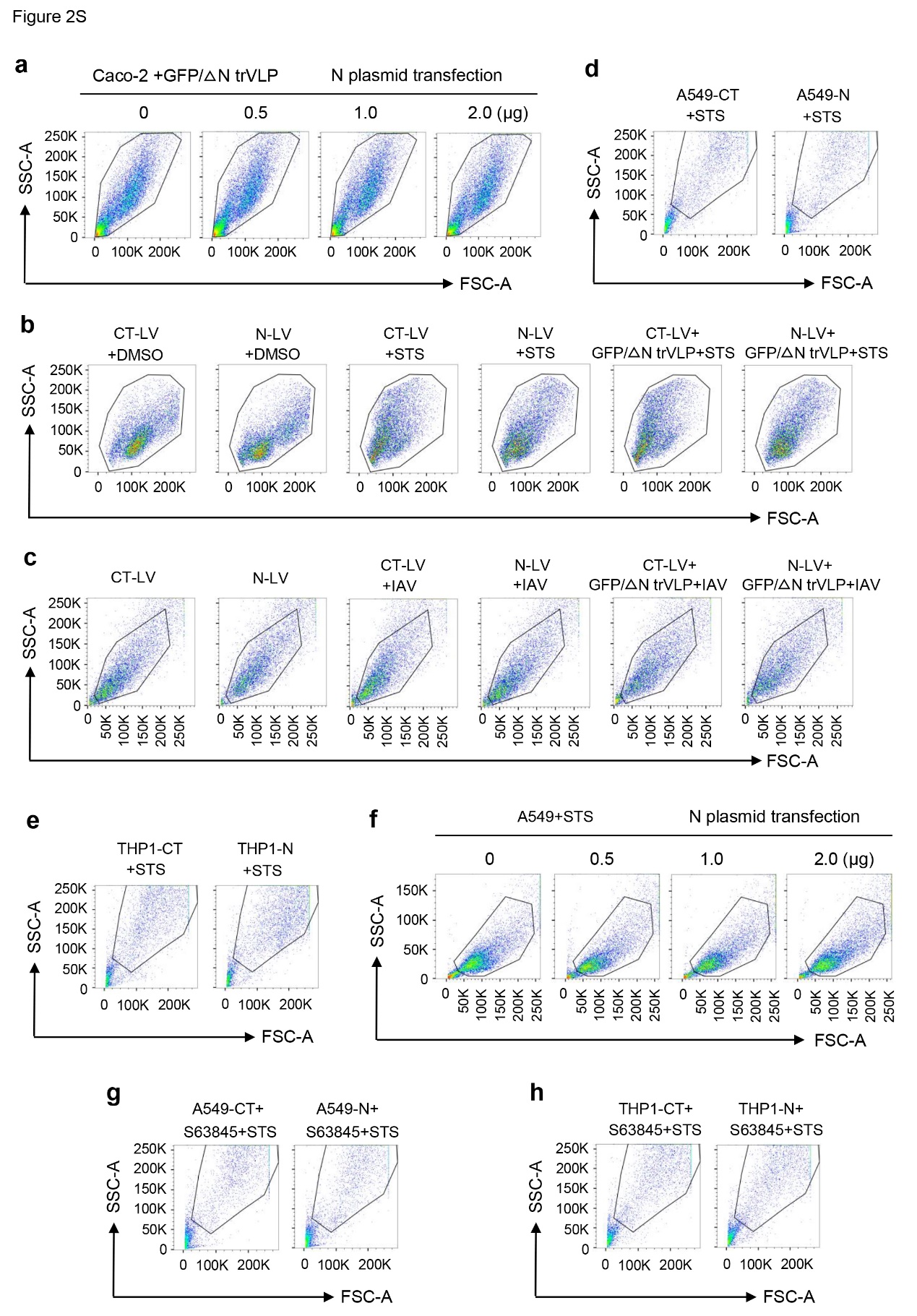


**Figure S2. Gating strategy for flow cytometry analysis. (a)** Caco-2 cells were transfected with different concentrations of plasmids encoding N for 24 h, and then infected with transcription and replication-competent SARS-CoV-2 virus-like-particles (SARS-CoV-2-trVLP) (MOI=0.5) for another 24h. Gating strategy for flow cytometry analysis. **(b)** Caco-2 cells were stably infected with Lentivirus-CT (CT-LV) or Lentivirus-N (N-LV), and CT-LV and N-LV cells pre-infected with SARS-CoV-2-trVLP (MOI=0.5) for 24h, then CT-LV and N-LV cells stimulated with 5 μM Staurosporine for another 4h. Gating strategy for flow cytometry analysis. **(c)** Caco-2 cells were stably infected with Lentivirus-CT (CT-LV) or Lentivirus-N (N-LV), and CT-LV and N-LV cells pre-infected with SARS-CoV-2-trVLP (MOI=0.5) for 12h, then CT-LV and N-LV cells infected with influenza virus (PR8 strain, MOI=0.1) for another 24h. Gating strategy for flow cytometry analysis. **(d, e)** A549 cells were stably infected with Lentivirus-CT or Lentivirus-N (d), THP-1 cells were stably infected with Lentivirus-CT or Lentivirus-N, differentiated into macrophages (e), and then stimulated with 5 μM Staurosporine or DMSO for 4 h. Gating strategy for flow cytometry analysis. **(f)** A549 cells were transfected with different concentrations of plasmids encoding N for 24 h, and then stimulated with 5 μM Staurosporine for 4 h. Gating strategy for flow cytometry analysis. **(g, h)** A549 cells (g) and THP-1 cells (h) were stably infected with Lentivirus-CT or Lentivirus-N. THP-1 cells were differentiated into macrophages. They were pre-treated with 3 μM MCL-1 inhibitor S63845 for 4 h, then stimulated with 5 μM Staurosporine or DMSO for 4 h. Gating strategy for flow cytometry analysis.

**Figure. S3**

**
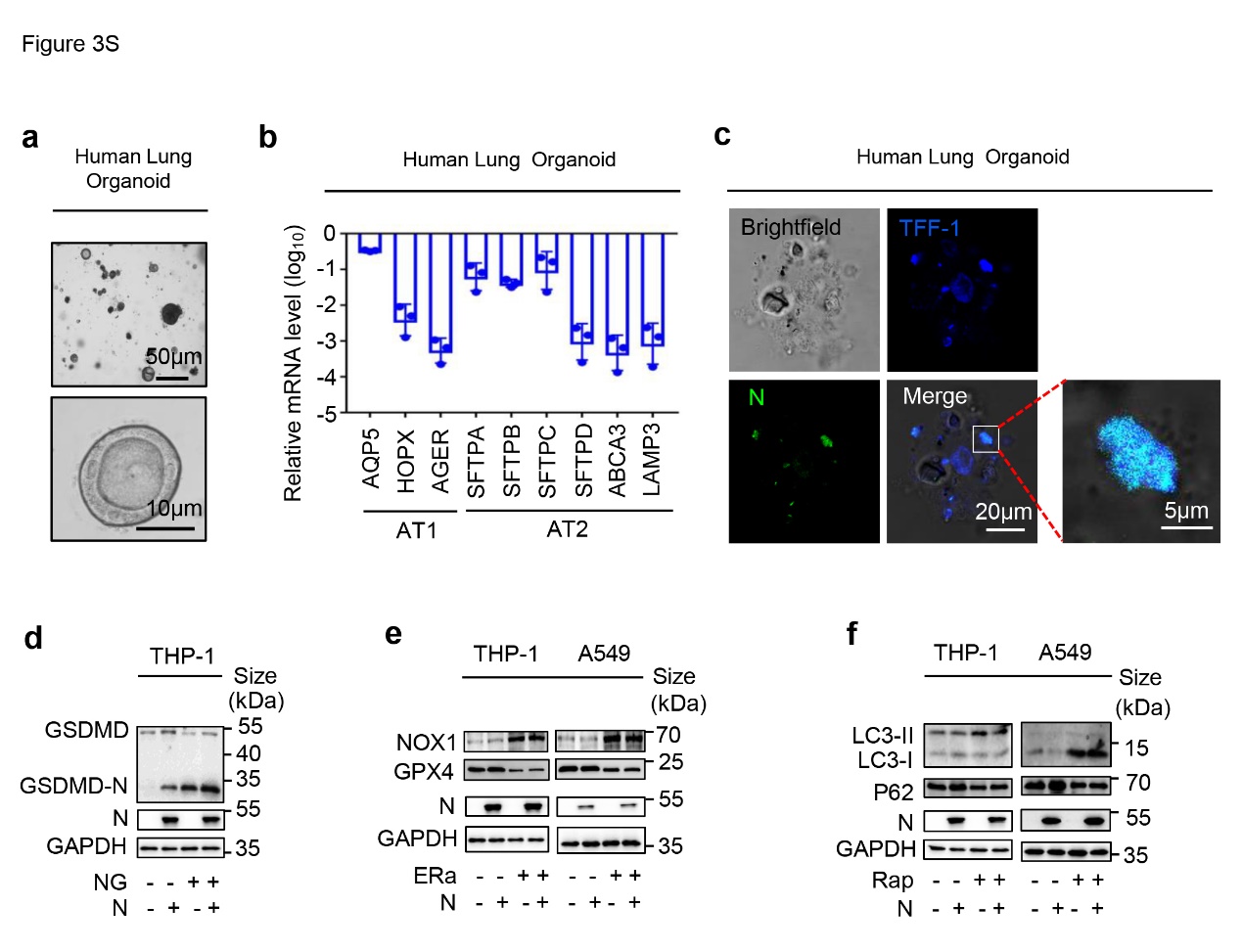
**

**Figure S3. Human lung organoids were constructed and N protein had influence in pyroptosis, autophagy and iron death.** (**a**) Human lung organoids were observed by using microscopy. Scale bar is 50 μm or 10μm. (**b**) Human lung organoids were collected, the mRNA levels of indicated genes were quantified by qRT-PCR. (**c**) Human Lung organoids were stably infected with Lentivirus-N, TFF-1 (blue) and Flag-SARS-CoV-2-N (green) were then visualized with confocal microscopy. Scale bar is 20 μm. **(d)** THP-1 cells were stably infected with Lentivirus-CT or Lentivirus-N, differentiated into macrophages, and then stimulated with 2 μM Nigericin or DMSO for 2 h. Cell lysates were analyzed by immunoblotting. **(e, f)** THP-1 cells were stably infected with Lentivirus-CT or Lentivirus-N, differentiated into macrophages, A549 cells were stably infected with Lentivirus-CT or Lentivirus-N, and then stimulated with 10 μM Erastin or DMSO for 4 h or stimulated with 500nM Rapamycin or DMSO for 24 h. Cell lysates were analyzed by immunoblotting. Data are representative of three independent experiments and one representative is shown. Data are representative of three independent experiments and one representative is shown.

**Figure. S4**

**
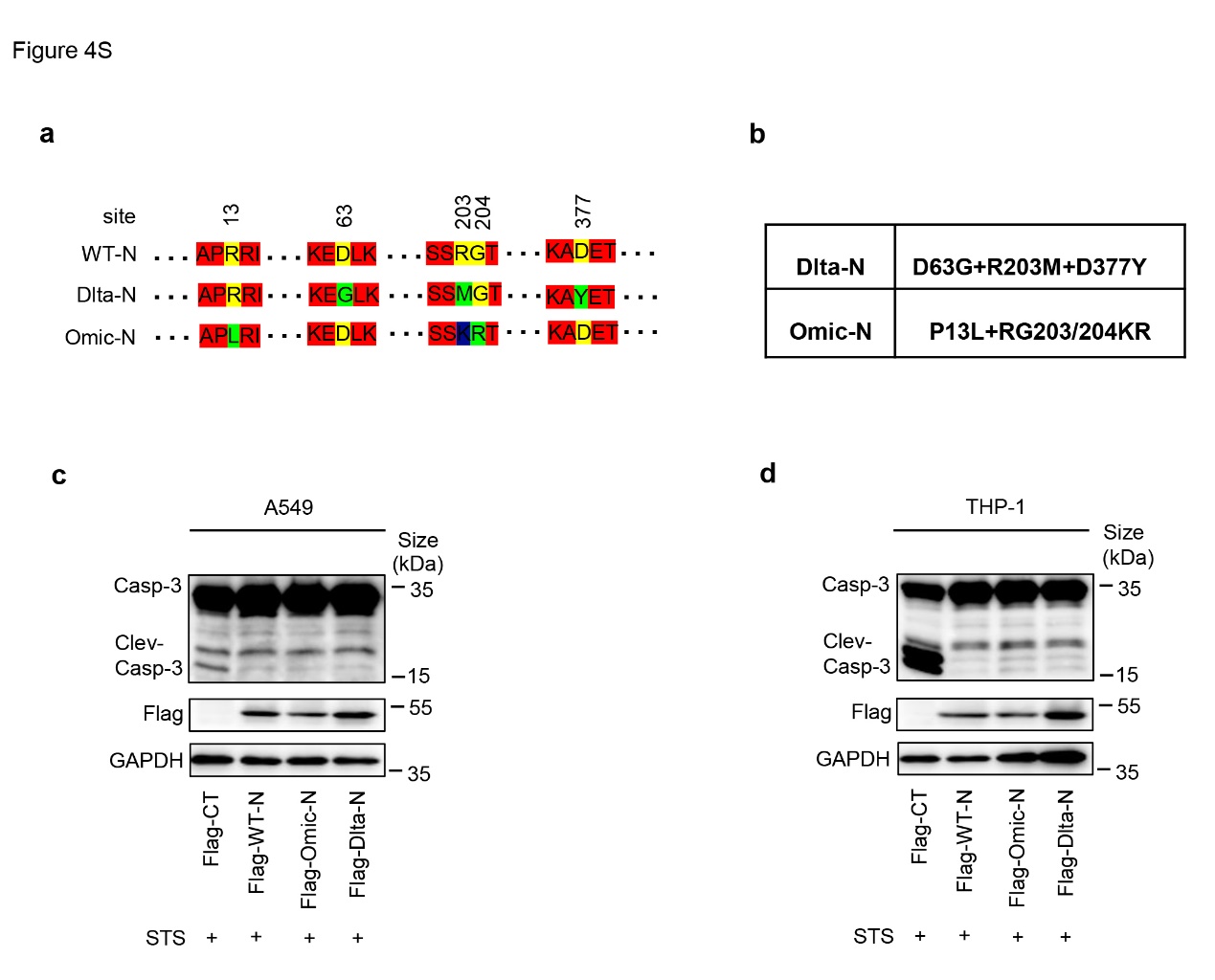
**

**Figure S4 SARS-CoV-2 Delta strain and Omicron strain N protein also inhibited cell apoptosis. (a)** N protein sequence comparison between original strain (WT) and mutant strains (Delta and Omicron). (b) Site-specific mutation of Delta and Omicron N protein. (c, d) A549 cells (c) or PMA-differentiated THP-1 macrophages (d) were respectively transfected with plasmids encoding WT-N, Omic-N and Dlta-N for 24 h, and then stimulated with 5 μM Staurosporine for another 4 h. Cell lysates were analyzed by immunoblotting. Flag-CT indicates pcDNA3.1(+)-3×flag empty plasmid (c, d). Data are representative of three independent experiments and one representative is shown.

**Figure. S5**

**
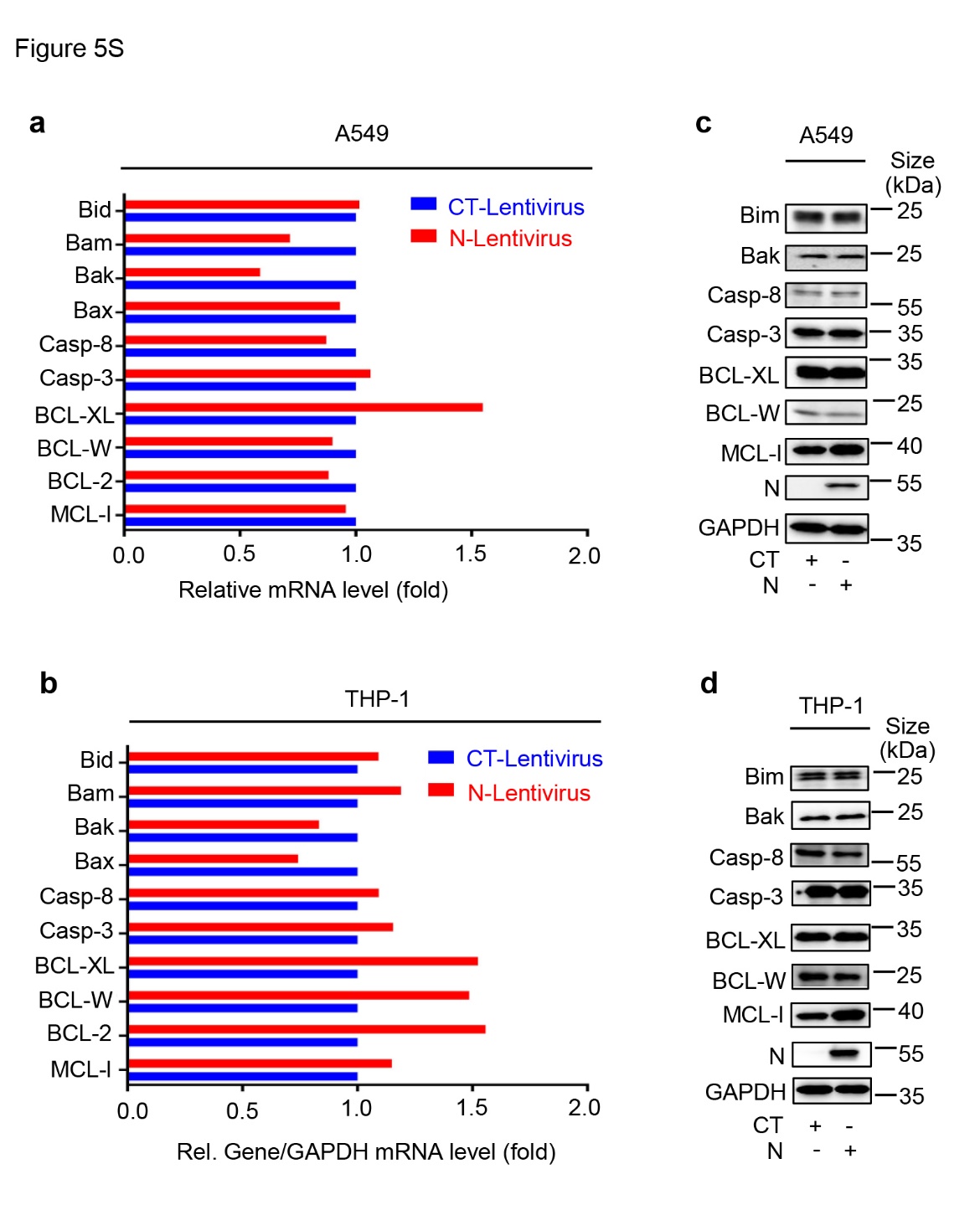
**

**Figure S5. N represses apoptosis by regulating MCL-1. (a-d)** A549 cells (a, c) or THP-1 cells (b, d) were stably infected with Lentivirus-CT or Lentivirus-N, THP-1 cells were differentiated into macrophages. The mRNA levels of indicated genes were quantified by qRT-PCR. (a, b). Cell lysates were analyzed by immunoblotting (b, d). CT means CT-lentivirus (c, d). Data are representative of three independent experiments and one representative is shown.

**Figure. S6**


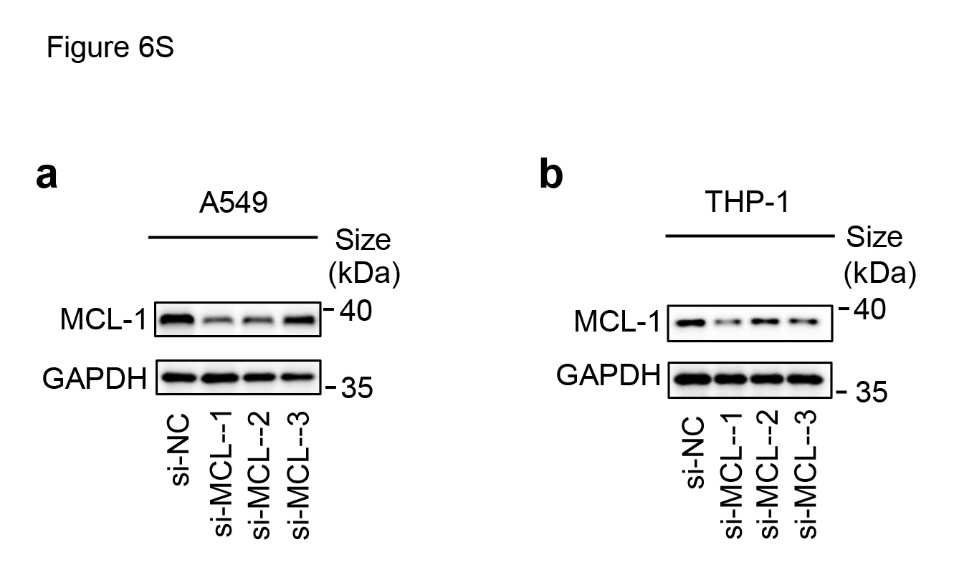


**Figure S6. Screening effectively knock-down the expression of MCL-1 by si-RNA. (a, b)** A549 cells (a) or PMA-differentiated THP-1 macrophages (c) were transfected with si-NC, si-MCL-1-1, si-MCL-1-2 or si-MCL-1-3 (50 nM), respectively, for 48h, and the cell lysates were analyzed by immunoblotting. Data are representative of three independent experiments and one representative is shown.

**Figure. S7**

**~~
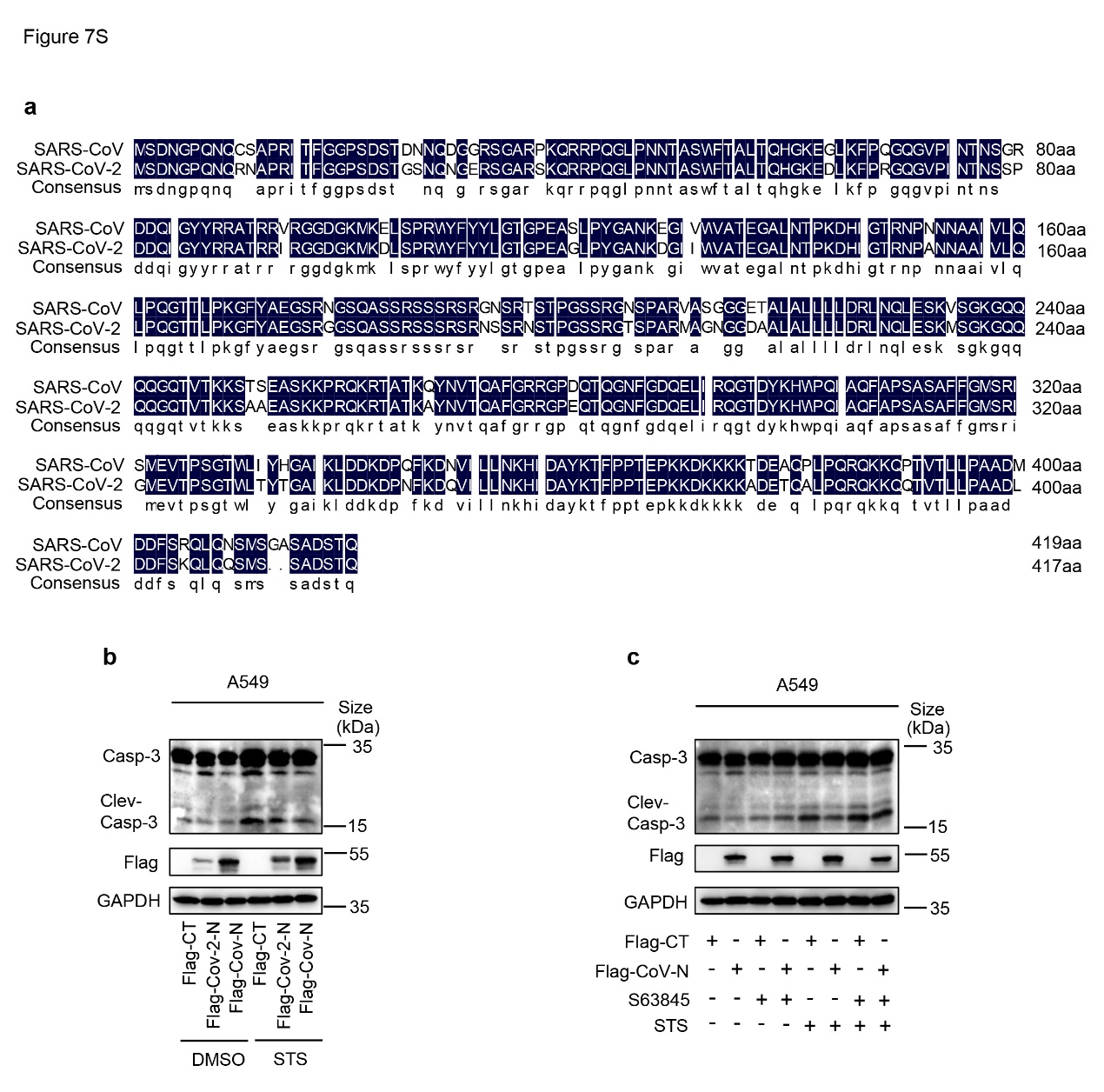
~~**

**Figure S7. SARS-CoV N protein also suppressed apoptosis by regulating MCL-1. (a)** N protein sequence comparison between SARS-CoV-2 and SARS-CoV. (b)A549 cells were respectively transfected with plasmids encoding CoV-2-N, and CoV-N for 24 h, and then stimulated with 5 μM Staurosporine or DMSO for another 4 h. Cell lysates were analyzed by immunoblotting. (c) A549 cells were transfected with plasmids encoding CoV-N for 24 h, they were pre-treated with 3 μM S63845 for 4 h, then stimulated with 5 μM Staurosporine or DMSO for another 4 h. Cell lysates were analyzed by immunoblotting. Flag-CT indicates pcDNA3.1(+)-3×flag empty plasmid (b, c). Data are representative of three independent experiments and one representative is shown.

**Figure. S8**


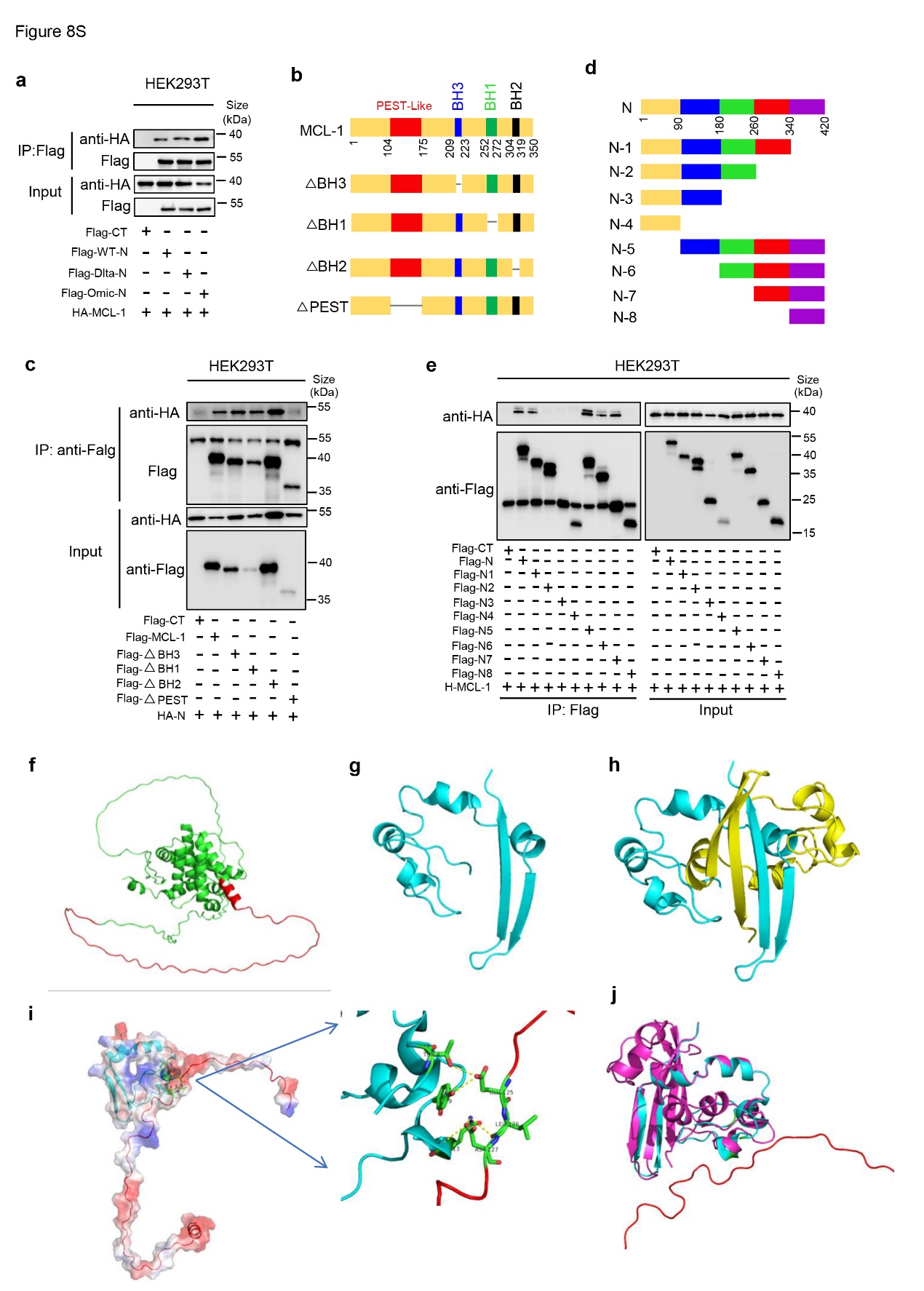


**Figure S8 SARS-CoV-2 N protein 260aa–340aa interacts with PEST-like domain of MCL-1 protein.** (a) HEK293T cells were co-transfected with HA-MCL-1 and Flag-CT, Flag-WT-N, Flag-Dlta-N or Flag-Omic-N for 24h. Cell lysates were immunoprecipitated using anti-Flag antibody, and analyzed using anti-Flag and anti-HA antibody. Cell lysates (40 μg) was used as Input. (b) Schematic diagram of wild-type MCL-1 protein and truncated mutants of MCL-1 protein (BH1, BH2, BH3 and PEST). (c) HEK293T cells were co-transfected with HA-SARS-CoV-2-N and Flag-ctrl, Flag-MCL-1, Flag-ΔBH3, Flag-ΔBH2, Flag-ΔBH1 or Flag-ΔPEST. Cell lysates were immunoprecipitated using anti-Flag antibody, and analyzed using anti-Flag and anti-HA antibody. Cell lysates (40 μg) was used as Input. (d) Schematic diagram of wild-type SARS-CoV-2-N protein and truncated mutants N protein (N1 to N8). (e) HEK293T cells were co-transfected with HA-MCL-1 and Flag-ctrl, Flag-N truncated mutants (N1-N8). (f) Predictive structure of MCL-1 protein. (g) Predictive monomer structure of N protein. (h) Predictive dimer structure of N protein. (i and j) Predictive complex structure of N and MCL-1 protein. Cell lysates were immunoprecipitated using anti-Flag antibody, and analyzed using anti-Flag and anti-HA antibody. Cell lysates (40 μg) was used as Input. Flag-CT means pcDNA3.1(+)-3×flag empty plasmid (a, c, e). Data are representative of three independent experiments and one representative is shown.

**Figure. S9**


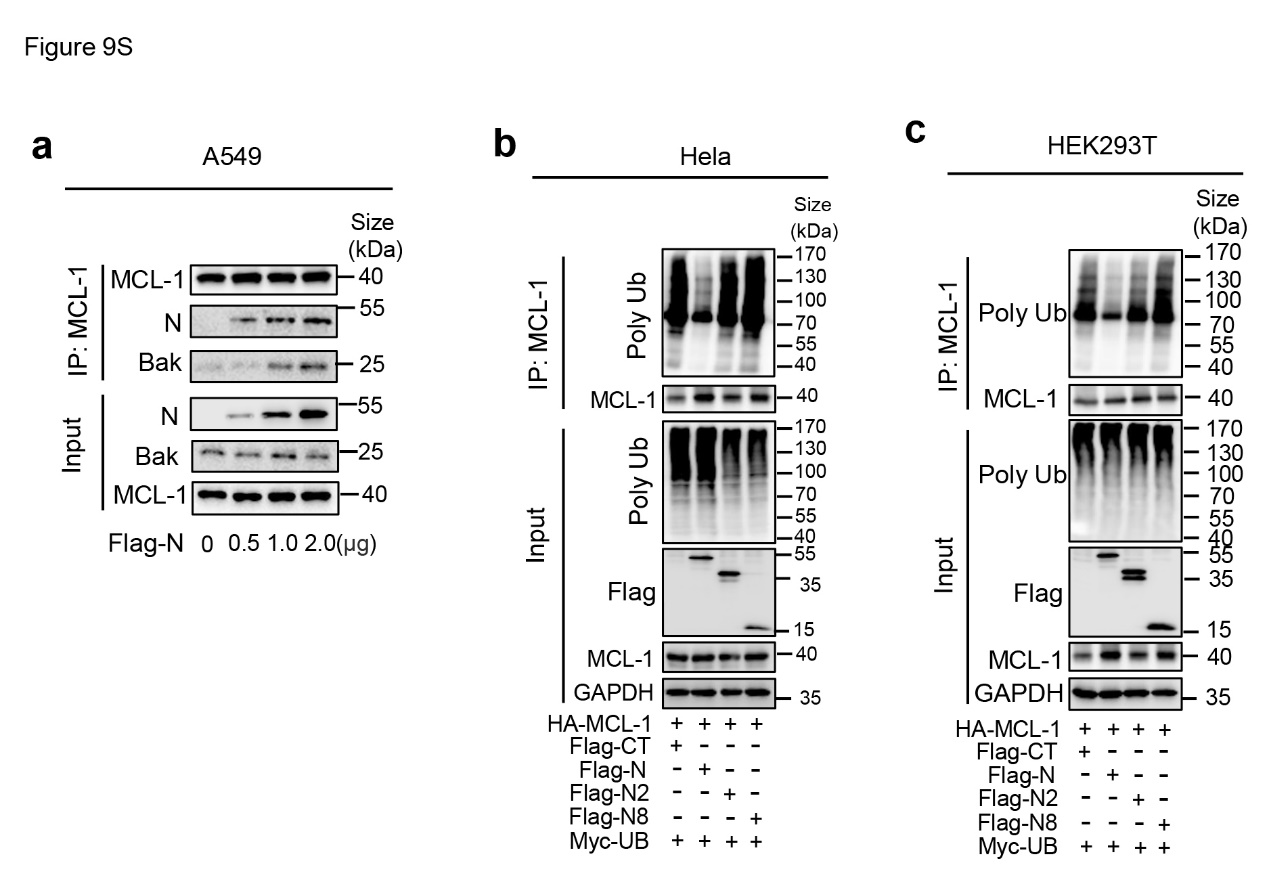


**Figure S9 SARS-CoV-2 N protein promotes the interaction between MCL-1 and Bak, or MCL-1 K63-linked deubiquitination. (a)** A549 cells were transfected with Flag-SARS-CoV-2-N at different concentration (0, 0.5, 1, 2 μg) for 24h. Cell lysates were immunoprecipitated using anti-MCL-1 antibody, and analyzed using anti-Bax, anti-N and anti-MCL-1 antibody. Cell lysates (40 μg) was used as Input. **(b, c)** Hela cells (b) or HEK293T cells (c) were co-transfected with HA-MCL-1, Flag-N, truncated mutants N protein (N2 and N8) or Myc-Ubiquitin for 24h. Cell lysates were immunoprecipitated using anti-HA antibody, and analyzed using anti-Myc, anti-Flag, anti-GAPDH and anti-HA antibody. Cell lysates (40 μg) was used as Input. Flag-CT means pcDNA3.1(+)-3×flag empty plasmid (b, c). Data are representative of three independent experiments and one representative is shown.

**Figure. S10**


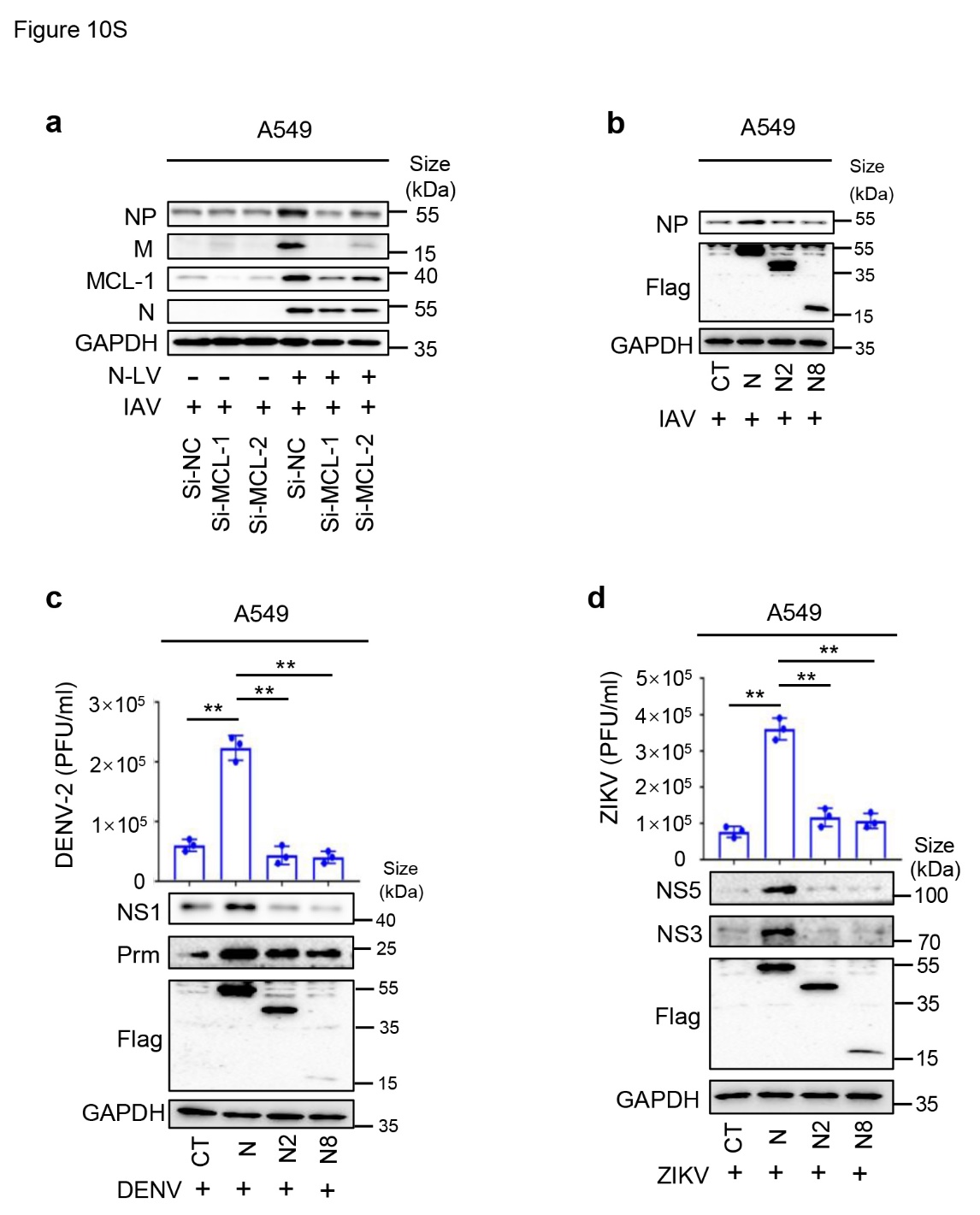


**Figure S10 SARS-CoV-2 N protein promotes RNA virus replication depending on MCL-1 protein.** (**a**) A549 cells were stably infected with Lentivirus-CT or Lentivirus-N, firstly transfected with si-NC, si-USP15-1 or si-USP15-2 (50 nM) for 24h, then infected with influenza virus (PR8 strain, MOI=0.1) for another 48h. Cell lysates were analyzed by immunoblotting. (**b**) A549 cells were firstly transfected with Flag-CT, Flag-N, truncated mutants N protein (N2 and N8) for 24h, then infected with influenza virus (PR8 strain, MOI=0.1) for another 48h, Cell lysates were analyzed by immunoblotting. **(c, d)** A549 cells were firstly transfected with Flag-CT, Flag-N, truncated mutants N protein (N2 and N8) for 24h, then infected with Dengue virus (NGC strain, MOI=0.5) (c) or ZIKV virus (PRV08 strain, MOI=0.5) (d) for another 48h, Cell supernatant were analyzed by plaque (upper), Cell lysates were analyzed by immunoblotting (lower). CT means pcDNA3.1(+)-3×flag empty plasmid (b-d). Data are representative of two independent experiments. and one representative is shown. Error bars indicate SD of each serum samples, P ≤0.05 (*), P ≤0.01 (**), P ≤0.001 (***), two-tailed Student’s t-test.

**Table S1**

**Table S1. qRT-PCR Primers used in this study**

| **Name** | **Forward** | **Reverse** |
| --- | --- | --- |
| Human  *MCL-1* | 5’- GACGAGTTGTACCGGCAGTC-3’ | 5’- TGATGTCCAGTTTCCGAAGCAT-3’ |
| Human  *BCL-2* | 5’- CTTTGAGTTCGGTGGGGTCA-3’ | 5’- GAAATCAAACAGAGGCCGCA-3’ |
| Human  *BCL-XL* | 5’- GCTTGGATGGCCACTTACCT-3’ | 5’- AAGAGTGAGCCCAGCAGAAC-3’ |
| Human  *BCL-W* | 5’- CACAGCTCTATACGGGGACG-3’ | 5’- AAGGCCCCTACAGTTACCAG-3’ |
| Human *GAPDH* | 5’-AAGGCTGTGGGCAAGG-3’ | 5’-TGGAGGAGTGGGTGTCG-3’ |
| Human  *Caspase-3* | 5’- CCTGGTTCATCCAGTCGCTT-3’ | 5’- TCTGTTGCCACCTTTCGGTT-3’ |
| Human  *Caspase-8* | 5’- GCTGACTTTCTGCTGGGGAT-3’ | 5’-GACATCGCTCTCTCAGGCTC-3’ |
| Human  *CCL2* | 5’-TGCAATCAATGCCCCAGTCA-3’ | 5’-GGGTCAGCACAGATCTCCTT-3’ |
| Human  *Bid* | 5’- AGGAGCACAGTGCGGATTC-3’ | 5’- TGCGGAAGCTGTTGTCAGAA-3’ |
| Human  *Bam* | 5’- GCCAGGCCTTCAACCACTAT-3’ | 5’- ACCATTCGTGGGTGGTCTTC-3’ |
| Human  *Bak* | 5’- AGGTTTTCCGCAGCTACGTT-3’ | 5’- TAGCGTCGGTTGATGTCGTC-3’ |
| Human  *Bax* | 5’- CATGGGCTGGACATTGGACT-3’ | 5’- AAAGTAGGAGAGGAGGCCGT-3’ |
| Human  *AQP5* | 5’- GCTCACTGGGTTTTCTGGGTA-3’ | 5’- TCCATGGTCTTCTTCCGCTC-3’ |
| Human  *HOPX* | 5’- GCCTTTCCGAGGAGGAGAC-3’ | 5’- TCTGTGACGGATCTGCACTC-3’ |
| Human  *AGER* | 5’- GTGTCCTTCCCAACGGCTC-3’ | 5’- ATTGCCTGGCACCGGAAAA-3’ |
| Human  *SFTPA* | 5’- GATGGGCAGTGGAATGACAGG-3’ | 5’- GGAATGAAGTGGCTAAGGGT-3’ |
| Human  *SFTPB* | 5’- GCAGACGTCCATAGCTCCTC-3’ | 5’- AGGGACACTTCCAGGCATTT-3’ |
| Human  *SFTPC* | 5’- AGCAAAGAGGTCCTGATGGA-3’ | 5’- CGATAAGAAGGCGTTTCAGG-3’ |
| Human  *SFTPD* | 5’- TGGGCTTCCAGATGTTGCTT-3’ | 5’- CGACACTTTGGCCATTTGGG-3’ |
| Human  *ABCA3* | 5’- AGATGTAGCGGACGAGAGGA-3’ | 5’- GCTGCTCGTACACCTTGGAG-3’ |
| Human  *LAMP3* | 5’- AAGATGACCACTTTGGAAATGTG-3’ | 5’-TGCGAGCAGCACGATTTAGA-3’ |
| Human  *USP15* | 5’- ATGGTGATGCCCAGTCACTT-3’ | 5’- TGTTCAACCACCTTTCGTGC-3’ |
| SARS-CoV-2  *M* | 5-GTGCCACTCCATGGCACTAT-3’ | 5’-TCCTTGATGTCACAGCGTCC-3’ |
| IAV-PR8  *NP* | 5- ATCACTCACTGAGTGACATC-3’ | 5-TCGTCCAATTCCACCAATCA-3’ |
